# Anticorrosive performance of newly synthesized dipyridine based ionic liquids by experimental and theoretical approaches

**DOI:** 10.1038/s41598-023-45822-9

**Published:** 2023-11-06

**Authors:** Amira Hossam Eldin Moustafa, Hanaa H. Abdel-Rahman, Mohamed Hagar, Mohamed R. Aouad, Nadjet Rezki, Sherif A. A. Bishr

**Affiliations:** 1 Chemistry Department, Faculty of Science, Alexandria University, P.O. 426 Ibrahemia, Alexandria, 21321 Egypt; 2 Faculty of Advanced Basic Sciences, Alamein International University, Alamein, Matrouh Governorate, Egypt; 3 Chemistry Department, College of Science, Taibah University, 30002 Al-Madinah Al-Munawarah, Saudi Arabia

**Keywords:** Chemical engineering, Electrochemistry

## Abstract

Two newly synthetic nontoxic dipyridine-based ionic liquids (P_ILs_) with the same chain lengths and different polar groups were investigated: bispyridine-1-ium tetrafluoroborate (**BPHP**, **TFPHP**) with terminal polar groups *Br* and *CF*_*3*_, respectively, on Carbon steel (CS) in 8M H_3_PO_4_ as corrosion inhibitors. Their chemical structure was verified by performing ^1^HNMR and ^13^CNMR. Their corrosion inhibition was investigated by electrochemical tests, especially as mass transfer with several characterizations: Scanning electron microscope/Energy dispersive X-ray spectroscopy (SEM–EDX), UV–visible, Atomic force microscope, Atomic absorbance spectroscopy, X-ray Photoelectron Spectroscopy and Gloss value. Theoretical calculation using density functional theory by calculating several parameters, molecular electrostatic potential, Fukui Indices, and Local Dual Descriptors were performed to demonstrate the reactivity behavior and the reactive sites of two molecules with a concentration range (1.25–37.5 × 10^–5^ M) and temperature (293–318 K). The maximum inhibition efficiency (76.19%) and uniform coverage were sufficient for **BPHP** at an optimum concentration of 37.5 × 10^–5^ M with the lowest temperature of 293 K. **TFPHP** recorded 71.43% at the same conditions. Two P_ILs_ were adsorbed following the El-Awady adsorption isotherm, including physicochemical adsorption. The computational findings agree with Electrochemical measurements and thus confirm CS's corrosion protection in an aggressive environment.

## Introduction

The damage by corrosion generates high inspection, repair, and replacement costs, constituting a public risk. Many techniques were explored to control this issue. Applying corrosion inhibitors has proven to be the most efficient method for increasing these materials' corrosion resistance and minimizing the harm they cause^1^. The corroding surfaces immediately become rough after the corrosion assault begins, significantly affecting the diffusion-controlled corrosion rate. Furthermore, when the generated corrosion layer is highly porous, surface roughness greatly increases the mass transfer rate of O_2_ to the electrodes of the electrochemical corrosion cell, resulting in higher corrosion rates. The most popular approach for controlling corrosion is the creation of novel chemicals that act as corrosion inhibitors, especially in acid media, due to several technical and financial benefits. The efficiency of these chemical compounds is affected by various factors, including temperature, humidity, shear stresses caused by fluid flow, and others. This kind of corrosion inhibitor is an organic chemical substance shielding the metallic substrate by generating an adsorbed layer that prevents water molecules and other corrosive species from reaching the surface^2^. The inhibition effectiveness depends on the ability of the inhibitor/surface system to form an adhesive and continuous layer and isolate the surface from the corrosive environment, thus decreasing the corrosion rate. The polar functional groups and the intermolecular interactions between the inhibitor molecules play a crucial role^3–5^. Based on the interaction strength between surface and inhibitor, the inhibitor compounds have been described by two kinds of interactions that corrosion inhibitors can use to adsorb at the interface: as being chemisorbed, which occurs when inhibitors and d-orbitals of the iron surface share electrons, and physisorbed, which occurs when charged molecules interact electrostatically with the metal surface.

Carbon steel (CS) is frequently used in various industries as the preferred construction material because it is less expensive than corrosion-resistant alloys. CS must be protected from corrosion because it has a broad scope of applications in industries, including power plants, the petroleum industry, and building materials, as it is cost-effective and imparts high mechanical strength^3, 6–8^. CS is frequently used as a structural component in industrial pipes, buildings, bridges, and kitchen appliances. CS is unstable chemically and reacts chemically or electrochemically with its surrounding environments to produce more stable corrosion products. In contrast, metal corrosion has always been a hot topic for researchers because of the risks to safety and the financial losses it causes to the industry, the way corrosion costs governments a lot of money^9^.

Acid solutions are generally used to remove undesirable scale and rust in several industrial processes. Because it is less corrosive than other mineral acids, Phosphoric acid (H_3_PO_4_) is a significant industrial chemical used as an intermediate in the fertilizer industry for metal surface treatment in the metallurgical industry. It is used for processes like chemical and electrolytic polishing or etching, chemical colouring, removing oxide film, phosphating, passivating, and surface cleaning. Despite its importance, it shows strong corrosiveness on CS. So, adding anti-corrosion materials slowed or inhibited the CS corrosion in phosphoric acid. Inhibitors are used in these processes to control metal dissolution as well as acid consumption^10–13^.

Therefore, adding an excellent inhibitor to an acidic environment is one of the best options. Many researchers are interested in the chemistry of ionic liquids (P_ILs_), which have collected extraordinary interest as powerful and have cautiously started on the more difficult challenge of industrial-oriented synthesis because they consider them corrosion inhibitors of green chemistry, thermal stable, have a strong adsorbent defence on the metal surface^14–19^. P_ILs_ have several advantageous physiochemical properties, including non-toxic, high conductivity, non-flammability, high thermal and chemical stability^19–25^, and environmentally friendly and non-hazardous due to their non-negligible vapor pressure. Additionally, P_ILs_ are highly soluble in polar corrosive environments due to their ionic nature. It is understandable that P_ILs_ with various cationic and/or anionic counters and show different side chains and cationic headgroups have been the interest of researchers in medicinal chemistry^26–32^, catalysis^33^, preventing certain metals and alloys from corroding^34^, electrochemistry^35^, biological activity in drug delivery and synthesis of drugs^36^, fuel production and processing^37, 38^, liquid crystal development^39^, biotechnology^40^, and other chemical processes. P_ILs_ are salts combining organic cations like pyridazinium, imidazolium, and pyrrolidinium with inorganic anions like chloride, bromide, and iodide. Pyridine is a toxic compound, but if it was developed with some additives and attached with different chains to be one of the P_ILs_ compounds, it approached pointing truly greener and more secure P_ILs_ to the environment.

Owing to rising environmental regulations, developing nontoxic alternatives compared to hazardous inorganic inhibitors is receiving more attention nowadays. Depending on their specific structural characteristics, they can exhibit diverse biological activities, synergistic effects and versatile applications. So, this paper is concerned with evaluating the action of the newly synthesized P_ILs_ on the electrochemical corrosion processes as anodic dissolution under mass transport control. Depending on its simplicity, the mass transfer coefficient can be obtained using the limiting current technique, which has gained wide acceptance in recent decades. The inhibitors were added with different concentrations at several temperatures to know their inhibition efficiency. Additionally, global quantum chemical descriptors, Fukui indices, local softness, local electrophilicity, dual Fukui function, dual local softness, and dual local philicity were investigated and discussed in more detail to understand the mechanism, the interactions, and the structural orientation of the two PILs and the metallic surface.

## Methodology (experimental)

### Synthesis of inhibitors molecules

**Figure 1:**
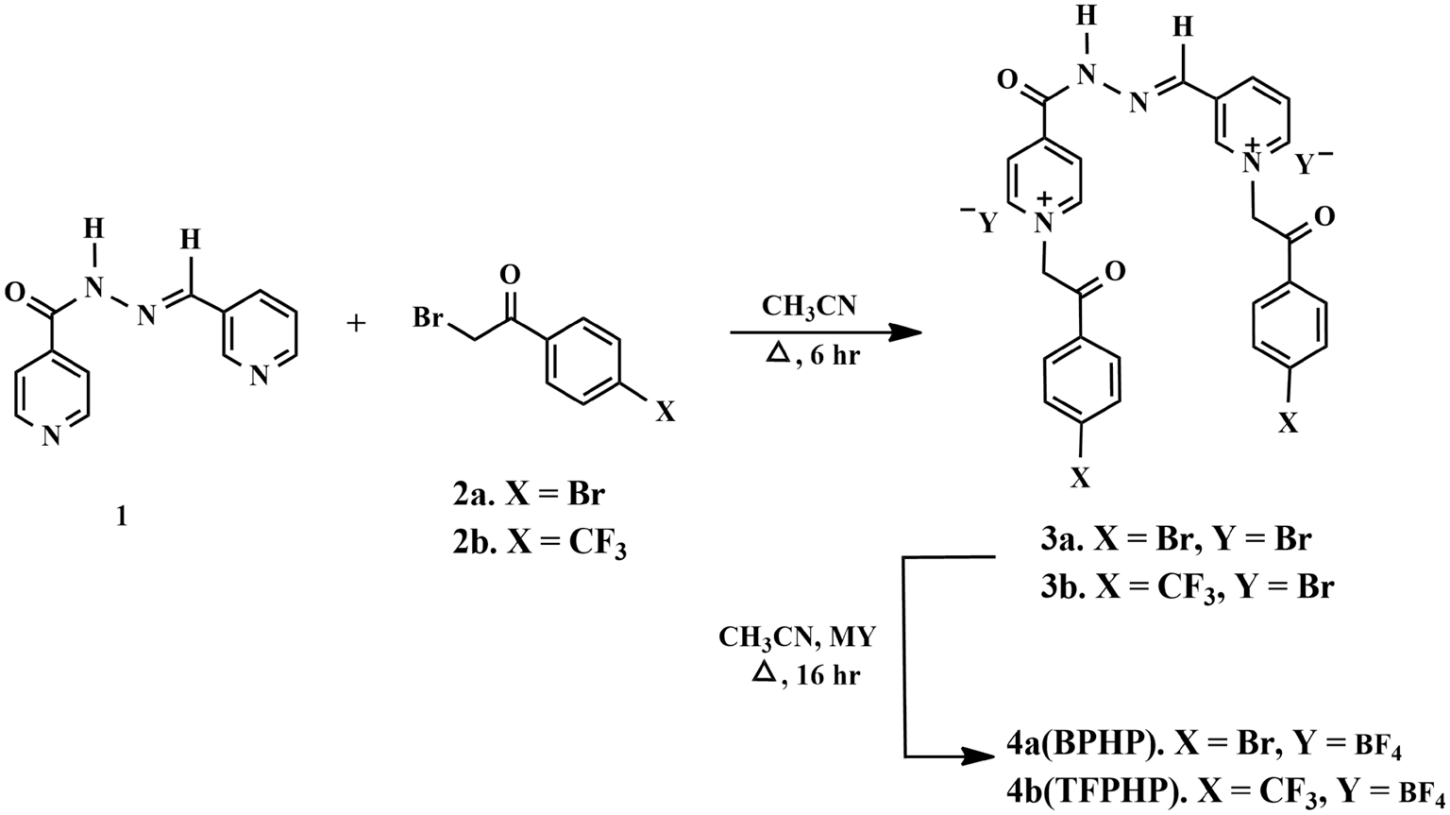
Synthetic routes for synthesizing di-cationic pyridinium ionic liquids** 4a–4b.**

According to our previous work, the desired (P_ILs_) 1-(2-(4-bromophenyl)-2-oxoethyl)-3-((2-(1-(2-(4-bromophenyl)-2-oxoethyl) pyridine-1-ium-4 carbonyl) hydrazono) methyl) pyridine-1-ium tetrafluoroborate (**BPHP**) and 1-(2-(4-trifluoromethyl phenyl)-2-oxoethyl)-3-((2-(1-(2-(4-trifluoromethylphenyl)-2-oxoethyl) pyridin-1-ium-4-carbonyl) hydrazono) methyl) pyridin-1-ium tetrafluoroborate (**TFPHP**) were prepared by quaternation of the pyridinium nitrogen atom of the generated bis-pyridine derivative^41^, followed by a metathesis event (Fig. 1).

Thus, the corresponding PILs **3(a, b)** were produced in good yield by thermally alkylating the initial bis-pyridine hydrazine **1** with two equivalents of 4-substituted-phenacyl bromide **2(a, b)** (Fig. 1). The ^1^H NMR, ^13^C NMR, and mass spectra analysis confirmed the synthesis of the intended **3a**. As a result, the two doublets at δ_H_ 6.67 ppm in the ^1^H NMR spectrum were assigned to the two new methylene protons (2×COCH_2_). An extra eight aromatic protons were also recorded at their usual chemical shifts belonging to the two aromatic rings of the phenacyl core. All remaining protons resonated in their respective area. The ^*13*^C NMR spectrum of **3a** supported the quaternization reaction, showing the methylene carbons resonating at δ_C_ 66.67 and 66.73 ppm, respectively.

The displacement of the bromide anion (Br^*−*^) and the furnishing of the desired task-specific PILs **4(a, b)** in good yield were accomplished by treating the synthesized dipyridinium bromide **3(a, b)** with potassium tetrafluoroborate. The success of the anion exchange was unambiguously evidenced by their spectroscopic results, which divulged that there was no change in their ^1^H and ^13^C NMR data compared to their corresponding starting pyridinium bromide **3(a, b)**. Correspondingly, the resulting P_ILs_ **4(a, b)** structure was deduced based on their ^31^P, ^11^B, ^19^F NMR, and mass spectra.

The structure of P_ILs_ **4 (a, b)** showed the presence of a multiple resonating between δ_B_ (−1.64) to (−1.78) ppm in its ^11^B spectrum and two doublets at δ_F_ −149.32 and −149.65 ppm in its ^19^F NMR spectrum respectively, supported the presence of tetrafluoroborate anion (BF_4_^−^) in the P_ILs_ **4 (a, b)** as counter anion, more spectral data of synthesis were mentioned in the Supplementary figures 1–6.

### Electrode sample and corrosive medium

The corrosive medium, 8 M H_3_PO_4_, was prepared using analytical grade phosphoric acid (85% w/w) supplied by Fisher Chemicals Ltd. The electrode sample used for electrochemical measurements was carbon steel (CS) having a surface area of 10 cm^2^ and the composition (wt.%) was determined by JEOL apparatus JSM-IT200 model: 0.14% Carbon, 0.02% Silicon, 0.56% Manganese, 0.03% Phosphorus, 0.01% Nickel, 0.01% Chrome, 0.01%Vanadium, 0.03% Aluminum, 0.04% Sulfur, and 99.15% Iron. We must be aware that the selection of 8 M *H*_*3*_*PO*_*4*_ is based on that the H_3_PO_4_ concentration effect on the value of limiting current *I*_*Lim*_ can be interpreted via the mass transform concept^10, 11^.

All experiments were carried out using 100 ml of the prepared phosphoric acid in various concentrations of the studied P_ILs_ inhibitors (1.25, 2.5, 7.5, 12.5, 17.5, 25 and 37.5) 10^–5^ M by dissolving in water for **BPHP** and **TFPHP**. The *I*_*Lim*_ was recorded for them at different temperatures: 293, 298, 308 and 318 K. Experiments were triple-checked to ensure the measurements were accurate and the results were within 2% error. The reported corrosion data is the average of the three measurements.

### Galvano-static polarization measurements

Due to its simplicity and accuracy, the Galvano-static technique may be a common tool to survey inhibitors' inhibition efficiency. Within the present study, standard methods that were described previously were used to perform the polarization experiments^10–13^.

### Spectroscopic analysis

After corrosion exposure by applying galvanic polarization, several characterizations were applied to CS in 8M H_3_PO_4_ without (blank) and with P_ILs_ inhibitors.

The quantity of Fe^2+^ was recorded by atomic absorption spectroscopy (AAS) estimated by ANALYTIK JENA CONTRAA 300 AAS to know the amount of iron ions passed into the solution after corrosion measurements and that for environmental protection.

The roughness of the CS surface was shown by atomic force microscope (AFM) Auto probe cp-research head manufactured by Thermomicrpscope operated in contact mode using Silicon Nitride probe model MLCT manufactured by Bruker. Proscan 1.8 software was used for controlling the scan parameters. Scan parameters: (contact mode, scan area 25 × 25 µm^2^, scan rate 1 Hz, number of data points 256 × 256 points) and IP 2.1 software for image analysis.

UV–visible reflectance spectroscopy (Jasco V-570) in the 200–700 nm range was used to get the brightness degree and reflection spectra.

Ultraviolet–visible absorption spectrophotometry [*Pg* instruments t-80 UV–Visible spectrophotometer] was used to recognize the absorption of metal and inhibitors in the solution.

Scanning electron microscope (SEM) shows the shielding layer on the CS surface and its morphological characterization. It coupled with the electron disperses X-ray spectroscopy (EDX) analyzer to determine the elemental constituents of layers formed on the corroded surface using a JEOL apparatus JSM-IT200 model.

X-ray photoelectron spectroscopy (XPS) analyses were performed by K-ALPHA (Themo Fischer Scientific, USA) with monochromatic X-ray AL K-alpha radiation (energy − 10 to 1,350 eV) under a vacuum of 10–9 with full-spectrum pass energy 200 eV at narrow-spectrum 50 eV. The analysis spot size was 400 μm in diameter. All binding energy values were determined concerning the C1s line originating from adventitious carbon.

### Theoretical studies

On the other hand, the theoretical calculations achieved the experimental data using Gaussian-09 with DFT results with method *B3LYP/6-311G (d, p)* showing the structures and electronic properties in the gas and liquid phase.

## Results and discussion

### Galvanostatic polarization curves

The electrochemical polarization experiments were conducted to understand the kinetics and suppression mechanism the studied PILs inhibitors provided at the *CS/H*_*3*_*PO*_*4*_ interface. This goal was provided by the analysis of current–potential curves obtained from Galvanostatic measurements.

**Figure 2:**
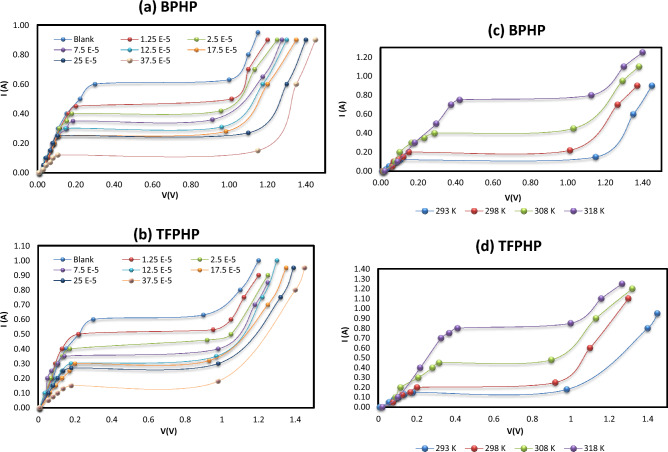
Polarization curves of the tested CS (**a**, **b**) at different P_ILs_ concentrations at 293 K & (**c**, **d**) at different temperatures for P_ILs_ concentration 37.5 × 10^–5^ M.

**Figure 3:**
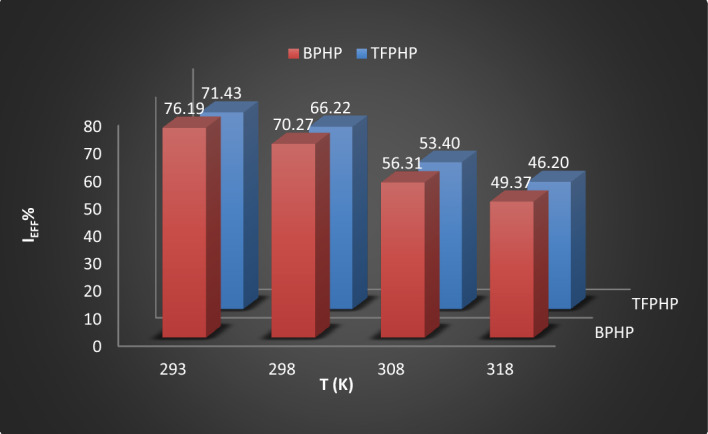
The effect of temperature on the inhibition efficiency (%${I}_{Eff}$) at constant concentration (37.5 × 10^–5^ M) of P_ILs_.

It can be seen from Fig. 2 and Table 1 that adding P_ILs_ compounds (**BPHP** & **TFPHP**) significantly reduced the corrosion limiting current values by increasing inhibitor concentrations and enhancements by temperatures compared to the blank curve. P_ILs_ inhibitors block the active sites of the CS surface, indicating that the corrosion of the iron electrode after the adsorption of P_ILs_ was more difficult, and two P_ILs_ showed excellent corrosion resistance. The values of inhibition efficiency (%${I}_{Eff}$) and the degree of the surface coverage was computed by the following Eq. (1)^13, 42^:

1 $$\% = \frac{{(I_{Lim} \left( {{\text{blank}}} \right) - { }I_{Lim} \left( {{\text{ILs}}} \right)}}{{I_{Lim} \left( {{\text{blank}}} \right)}} \times { 1}00$$

where I_Lim_(blank) and I_Lim_(P_ILs_) are the limiting currents without and with a concentration of inhibitors, respectively. As seen in Table 1, the %${I}_{Eff}$ were enhanced with increasing inhibitor concentration to reach 76.19–71.43% for 37.5 × 10^–5^ M of **BPHP** and **TFPHP**, respectively. By the way, when adding a higher concentration of the inhibitor than 37.5 × 10^–5^ M to the solution, the inhibitor would be desorbed from the surface of the metal. Even though, due to the desorption of the inhibitor molecules by increased temperatures, the ${I}_{Eff}$ of P_ILs_ decrease from 76.19 to 46.20% in the case of temperature variation. This may be attributed to the high dissolution rates of CS at elevated temperatures due to increased solution agitation resulting from the high rate of *H*_*2*_ gas evolution. This may also reduce the ability of inhibitors to be adsorbed on the CS surface. The results tabulated indicate that the investigated molecules work by adhering to the CS surface and assisting in forming an inhibitory layer that serves as a barrier between the CS surface and the corrosive media's constituent parts. Since **BPHP** has a bigger ${I}_{Eff}$ than **TFPHP** and a lower desorption rate than **TFPHP**; it is a better inhibitor than the other one (Fig. 3).

**Table 1:** Limiting current (I_lim_) values and inhibition efficiency (%${I}_{Eff}$) with and without P_ILs_ at different temperatures and concentrations.

P_ILs_	C_PILs_ × 10^5^ (M)	293 K		298 K		308 K		318 K	
		I_lim_	% I_Eff_	I_lim_	% I_Eff_	I_lim_	% I_Eff_	I_lim_	% I_Eff_
BPHP	Blank	0.63	0.00	0.74	0.00	1.03	0.00	1.58	0.00
	1.25	0.50	20.63	0.62	16.22	0.90	12.62	1.44	8.86
	2.50	0.42	33.33	0.55	25.68	0.80	22.33	1.27	19.62
	7.50	0.36	42.86	0.47	36.49	0.69	33.01	1.10	30.38
	12.50	0.31	50.79	0.41	44.59	0.61	40.78	0.97	38.61
	17.50	0.28	55.55	0.37	50.00	0.56	45.63	0.89	43.67
	25.00	0.27	57.14	0.34	54.05	0.51	50.48	0.83	47.47
	37.50	0.15	76.19	0.22	70.27	0.45	56.31	0.80	49.37
TFPHP	Blank	0.63	0.00	0.74	0.00	1.03	0.00	1.58	0.00
	1.25	0.53	15.87	0.64	13.51	0.91	11.64	1.47	6.96
	2.50	0.46	26.98	0.57	22.97	0.81	21.36	1.34	15.19
	7.50	0.40	36.51	0.49	33.78	0.73	29.12	1.21	23.42
	12.50	0.35	44.44	0.43	41.89	0.64	37.86	1.06	32.91
	17.50	0.32	49.21	0.39	47.30	0.57	44.66	0.96	39.24
	25.00	0.30	52.38	0.36	51.35	0.54	47.57	0.92	41.77
	37.50	0.18	71.43	0.25	66.22	0.48	53.40	0.85	46.20

### Adsorption isotherm behavior and thermodynamics parameters

It would be precious to make sense of the adsorption processing by a fitting adsorption isotherm, which could give further helpful bits of knowledge into the association of the inhibitor with the metal surface and, subsequently, the system of corrosion restraint. To decide the best adsorption isotherm model, which improves the degree of surface coverage area (*θ*) upon the CS surface. As could be seen, the formation of a defensive layer over the metallic surface, which reduces the reactive surface area for corrosive ion adhesion and hence mitigates corrosion, was shown as the inhibitor quantity was increased by the increase in the *θ* across the CS substrate. The direct types of considered adsorption isotherm models are as the following: Langmuir, El-Awady and Flory–Huggins applied for the studied P_ILs_ (**BPHP** &**TFPHP**), were determined at 298 K for different concentrations. The direct types of considered adsorption isotherms are described in Table 2, where $\theta$ = surface coverage = ${I}_{Eff}$%, ${C}_{{P}_{ILs}}$ is the P_ILs_ concentration, *y* is the number of inhibitor molecules involving one dynamic site, *x* is the number of water atoms supplanted by one particle of the inhibitor, and *K*_*ads*_ is the constant of equilibrium of the adsorption interaction, which is temperature dependent. It is related to the free energy of adsorption according to Eq. (2):

2 $$\Delta G_{ads} = - RTln \, \left( {55.5 \, K_{ads} } \right)$$

**Table 2:** Linear isotherm equations and adsorption parameters for P_ILs_ at 298 K.

Isotherm model	Linear form	Plot	Parameters	Inhibitors	
				BPHP	TFPHP
Langmuir	$\frac{{C}_{{P}_{ILs}}}{\uptheta }$= $\frac{1}{{\mathrm{K}}_{\mathrm{ads}}}$+${C}_{{P}_{ILs}}$	$\frac{{C}_{{P}_{ILs}}}{\uptheta }$ vs ${C}_{{P}_{ILs}}$	Slope	1.31	1.36
			R^2^	0.9578	0.9623
El-Awady	$\mathrm{log}\left(\frac{\uptheta }{1-\uptheta }\right)={\mathrm{logK}}^{^{\mathrm{\prime}}}+\mathrm{ylog}{C}_{{P}_{ILs}}$ (K = ${\mathrm{K{\prime}}}^{\frac{1}{\mathrm{ y}}}$)	$\mathrm{log}\left(\frac{\uptheta }{1-\uptheta }\right)\mathrm{ vs log}{C}_{{P}_{ILs}}$	R^2^	0.9663	0.9779
			y	0.68	0.68
			1/y	1.47	1.48
			K$\mathrm{^{\prime}}$	402.90	337.36
			K_ads_	6921.69	5451.00
			∆G_ads_ (kJ/mol)	−31.86	−31.27
Flory–Huggins	$\mathrm{log}\left(\frac{\uptheta }{{C}_{{P}_{ILs}}}\right)={\mathrm{logxK}}_{\mathrm{ads}}+\mathrm{xlog}(1-\uptheta )$	$\mathrm{log}\left(\frac{\uptheta }{{C}_{{P}_{ILs}}}\right)\mathrm{ vs log}(1-\uptheta )$	R^2^	0.9838	0.9823
			x	3.05	2.98
			K_ads_	4673.35	4170.24
			∆G_ads_ (kJ/mol)	−30.89	−30.60

**Figure 4:**
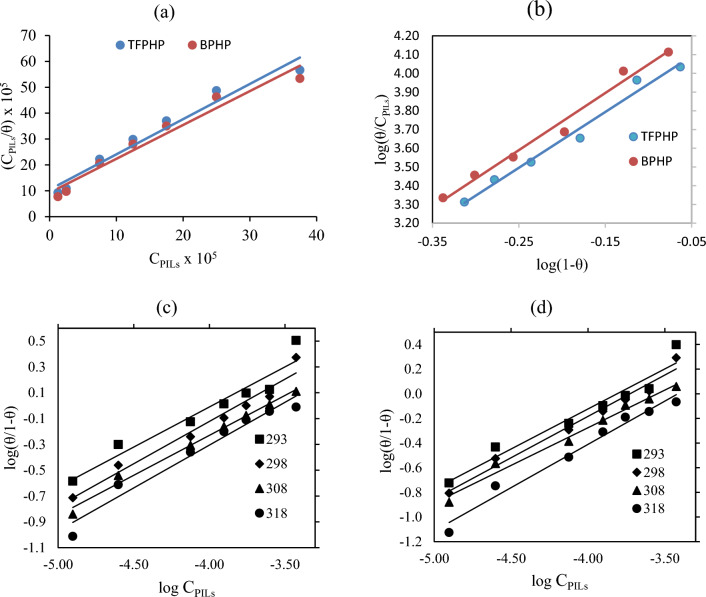
P_ILs_ adsorption isotherm models for CS tested electrodes in 8 M H_3_PO_4_ with different concentrations for isotherm equations in Table 2 (**a**) Langmuir model by plotting (**b**) Flory–Huggin’s model. Elawady model (**c**) **BPHP** (**d**) **TFPHP.**

*R*,* T*, and 55.5 M are the ideal gas constant, work temperature (293–318 K), and water content, respectively. Figure 4 shows the linear fit according to the curve derived from the Galvanostatic technique. The adsorption parameters deduced from these isotherms and values of the determination coefficient (*R*^2^) are regrouped in Table 2.

Figure 4a represents Langmuir’s adsorption relationship with linear correlation coefficient (*R*^2^) values approaching one. The outcomes indicate that the Langmuir model displays the best linear relationship, but the slope deviates markedly from unity for **BPHP** and **TFPHP**^43, 44^. Besides, the Langmuir model did not achieve; it remains hypothetical and often referred to as ideal adsorption, which is not really applicable in concrete complex electrochemical systems. Electrochemical systems are often referred to as either ideal or non–ideal adsorptions because of the different assumptions on which each adsorption isotherm is based. So, we were obliged to test other isotherm models, namely El–Awady and Flory–Huggins.

It should be pointed out that El–Awady and Flory–Huggin’s isotherm can be used to examine the number of water molecules that can be replaced by one inhibitor molecule on the CS surface.

As shown in Fig. 4b, the linear form of Flory Huggins adsorption isotherm as log *θ/C*_PILs_ vs log (*1−θ*) at 298 K. The obtained data reported in Table 2 yielded a linear correlation coefficient (*R*^2^) with slope (*x*) and intercept (*xK*). We found that the calculated values of (*x*) were higher than unity, implying that one **BPHP** and **TFPHP** molecule replaces more than a water molecule at a constant temperature.

On the other hand, the curve fitting of the El-Awady model is shown in Fig. 4c, d at different temperatures, and the calculated values of *K, K′* and *1/y* are listed in Table 3. The strong correlations confirm the validity of the approach. The values of *1/y* ensure that every molecule in the adsorbent mechanism is linked to greater than one active site on the CS outermost layer. It is confirmed that the corrosion inhibitor forms a dense multilayer physisorption of P_ILs_ molecules on the CS surface.

**Table 3:** Thermodynamic parameters data of P_ILs_ by ELAwady adsorption model.

P_ILs_	BPHP							TFPHP						
T (K)	y	1/y	K'	K_ads_	∆G_ads_ (kJ/mol)	∆H_ads_ (KJ/mol)	∆S°_ads_ (J/ mol.K)	y	1/y	K'	K_ads_	∆G_ads_ (kJ/mol)	∆H_ads_ (KJ/mol)	∆S°_ads_ (J/ mol.K)
293	0.62	1.61	297.17	9628.71	−32.13	−35.46	−12.08	0.68	1.47	422.28	7049.53	−31.37	−34.33	−10.29
298	0.68	1.47	402.90	6921.69	−31.86			0.68	1.48	337.36	5451.00	−31.27		
308	0.64	1.57	211.88	4495.68	−31.82			0.61	1.64	147.67	3541.99	−31.21		
318	0.51	1.97	58.36	2996.22	–31.78			0.61	1.64	112.07	2307.51	-31.09		

Table 2 shows that the values of *K*_*ads*_ obtained from the El-Awady model were large compared to Flory Huggins’s adsorption isotherm. In addition, the large value of *K*_*ads*_ indicates the high adsorptive power of **BPHP**. On the other hand, Table 3 shows the high values of *K*_*ads*_ observed at low temperatures mean that strong interactions of the P_ILs_ molecules with the *Fe* were favored at this temperature.

The free energy of adsorption *(∆G*_*ads*_) values are assigned from *K*_*ads*_ values of the El-Awady adsorption model, which showed the best correlation with the experimental data. The computed values of *∆G*_*ads*_ for the P_ILs_ compounds at various temperatures are recorded in Table 3. Negative signs of *∆G*_*ads*_ elucidate the spontaneous adsorption of the studied P_ILs_ on the CS surface ^45^. The *∆G*_*ads*_ values range from −32.13 to –31.78 kJ.mol^-1^ for **BPHP** and from −31.37 to −31.09 kJ. mol^-1^ for **TFPHP,** clarifying that the P_ILs_ adsorption process in 8M *H*_*3*_*PO*_*4*_ involves both physisorption and chemisorption mechanisms (physicochemical), which signifies a complex mode between CS and inhibitor molecules^46–49^. In another way, physicochemical meaning involves electrostatic interactions between the charged molecules and the metal (physisorption) and also sharing or transfer of electron pairs or π electrons from organic molecules to the metal surface to form a coordinate bond (chemisorption).

Else important thermodynamic parameters are obtained by Vant’t Hoff equation, which is utilized to assess enthalpy of adsorption (*∆H*_*ads*_) by plotting *lnK*_*ads*_ vs *1/T*. A linear relation is gained with a slope equal to (−*∆H*_*ads*_) /*R*). The values of (*∆H*_*ads*_) were computed and registered in Table 3. The negative amount of *∆H*_*ads*_ shows that the adsorption of the inhibitor is an exothermic interaction; this outcome might make sense of how the adsorption is consistent, and afterward, the inhibition decreases with expanding temperature. In this work, the values of *∆H*_*ads*_ for the adsorption of the inhibitor are − 35.46 kJ/mol for **BPHP** and −34.33 kJ/mol for **TFPHP**. This value is closer to − 40 kJmol^−1^ and far from − 100 kJmol^−1^. It implies that the adsorption of P_ILs_ follows physicochemical^50^. The Gibbs–Helmholtz equation is utilized to deduce the standard entropy of the adsorption (*∆°S*_*ads*_) at 298 K according to the following Eqs. (3) and (4):

3 $$lnK_{ads} = \left( {\frac{{ - \Delta H_{ads} }}{RT}} \right) + Constant$$

4 $$\Delta G_{ads}^{\circ} = \Delta H_{ads}^{\circ} - T\Delta S_{ads}^{\circ}$$

The negativity of adsorption entropy *∆S°*_*ads*_*,* obtained in this work, suggests a reduction in the translational degrees of freedom and perturbation, possibly due to the accumulation of water molecules on the surface.

**Figure 5:**
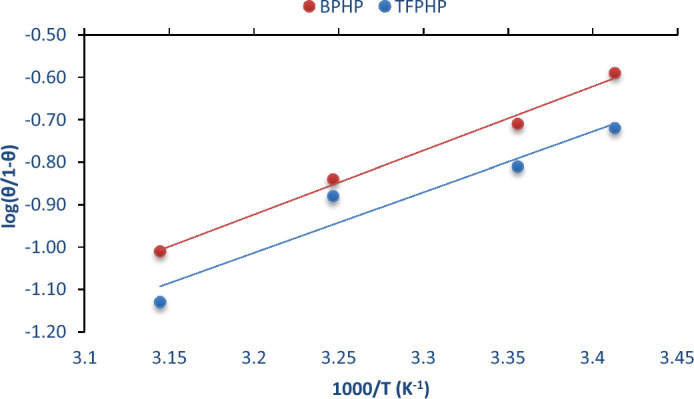
The active thermodynamic model used for driving the heat of adsorption (*Q*_*ads*_).

The active thermodynamic model used for driving the heat of adsorption (*Q*_*ads*_) for extra increased understanding of the adsorption procedure using Eq. (5)^51^:

5 $$\log \left( { \frac{\theta }{1 - \theta }} \right) = \log A + \log C_{P_{ILs} } - \left( {\frac{{Q_{ads} }}{2.303RT}} \right)$$

where *A* is a constant and ${C}_{{P}_{ILs}}$ is the concentration of inhibitors, Fig. 5 shows the relation between *log* $\left(\frac{\theta }{1-\theta }\right)$ with $\frac{1000}{T}$, the slope equals $-(\frac{Q}{2.303R})$. For the **BPHP** (*Q*_*ads*_ = $-$ 28.872 kJ/mol) and **TFPHP** (*Q*_*ads*_ = $-$ 27.349 kJ/mol). The negative value of *Q*_*ads*_ shows that the surface coverage level diminishes with a temperature rise in the presence of inhibitors and exothermic processes^52, 53^. The higher absolute value of **BPHP** means that its molecules were adsorbed more than **TFPHP**.

To recognize physisorption and chemisorption, the isotherm of Dubinin-Radushkevich has been utilized and described as follows^54^:

6 $${\text{ln}}\theta = {\text{ ln}}\theta_{{\text{max}}} - {\text{a}}\delta^{2}$$

where θ_max_ is the maximum surface coverage, and δ is the Polanyi potential described by:

7 $$\delta = RTln\left( {1 \, + \frac{1}{{C_{P_{ILs} } }}} \right)$$

**Figure 6:**
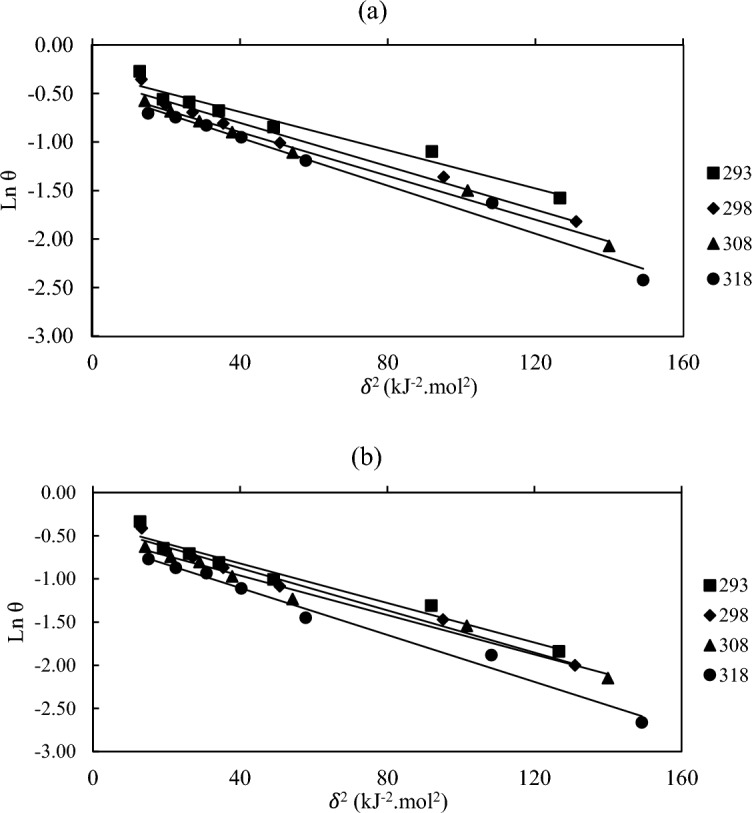
Dubinin-Radushkevich isotherm to recognize physical or chemical adsorption (**a**) **BPHP** (**b**) **TFPHP.**

By plotting *ln θ* against δ^2^ Fig. 6, the constant *a* was obtained from the slope. The values of *a* prompt the mean adsorption energy *E*_*m*_ for the different temperatures are in Table 4 This energy, which is the exchange energy of 1 mol of adsorbate from the solution bulk to the outer layer of the adsorbent, is characterized as:

8 $$E_m = \frac{1}{{\sqrt {2a} }}$$

**Table 4:** Data of Dubinin-Radushkevich adsorption energy isotherm to recognize physical or chemical adsorption.

P_ILs_	T (K)	R^2^	a (kJ^−2^ mol^2^)	θ_max_	E_m_ (kJmol^−1^)
BPHP	293	0.9563	0.0099	0.74378	7.1066905
	298	0.9734	0.0112	0.70082	6.681531
	308	0.9912	0.0113	0.63979	6.65190
	318	0.9797	0.0124	0.63027	6.35001
TFPHP	293	0.9604	0.0115	0.69607	6.59380
	298	0.9796	0.0122	0.67997	6.40184
	308	0.9815	0.0114	0.60429	6.62266
	318	0.9854	0.0136	0.57103	6.06339

The extent of *E*_*m*_ gives data about the kind of adsorption type to be chemisorption or physisorption: *E*_*m*_ values under 8 kJmol^-1^ demonstrate physical adsorption, while those higher than 8 kJmol^−1^ recommend chemisorption, so the *E*_*m*_ values mean physical adsorption for the two inhibitors^54–57^.

### Kinetic parameters

The kinetic model was another helpful tool for making sense of the erosion resistance and further explaining the inhibitors' features. The activation energy values *E*_*a*_ were evaluated via the linearized form of the Arrhenius equation with a temperature range of (293–318 K) for CS disintegration in the absence and presence of **BPHP** and **TFPHP** as follows:

9 $$lnI_{Lim} = \, lnA E_a /RT$$

**Figure 7:**
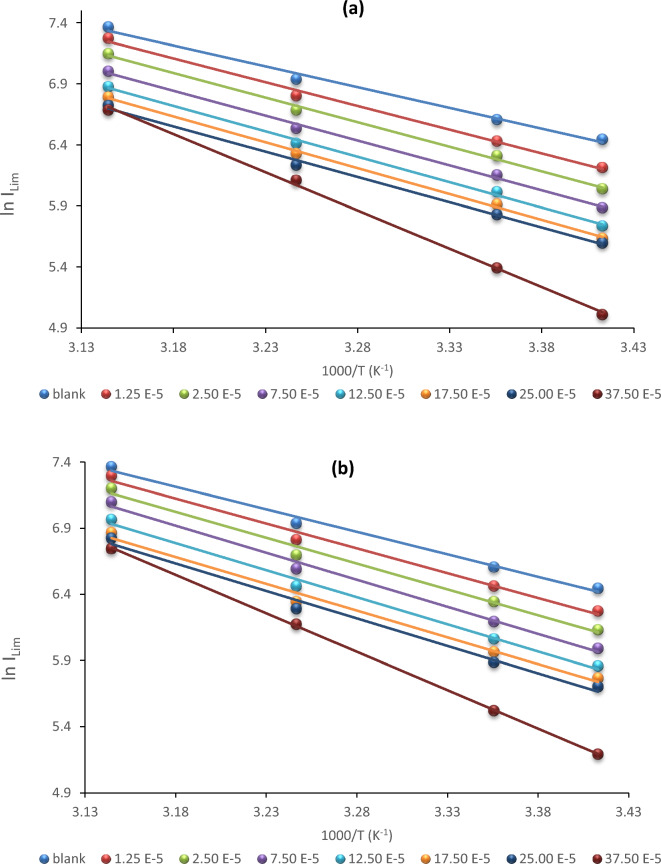
Arrhenius graphs for determination energy of activation from the relation between limiting current and temperature with and without P_ILs_ (**a**) **BPHP** (**b**) **TFPHP.**

The linear regression plots between ln *I*_*Lim*_ versus 1000/T are presented in Fig. 7; the values of *E*_*a*_ (energy of activation) are derived from the slopes = ( *− E*_*a*_*/R*) where gas constant *R* = 8.314 JK^−1^ mol^−1^ and *A* is that the factor of frequency. The calculated data at different inhibitor concentrations were collected in Table 5. The change in the values of the apparent activation energies may be explained by the corrosion process's mechanism alterations in the presence of adsorbed P_ILs_ inhibitor molecules.

**Table 5:** The activation parameters values *E*_*a*_*, ∆H*^*≠*^*, ∆S*^*≠*^* and ∆G*^*≠*^ with and without P_ILs_ of different concentrations in 8 M *H*_*3*_*PO*_*4*_.

P_ILs_	C_PILs_ × 10^5^ M	E_a_ kJ/ mol	∆S^**≠**^ J/mol.K	∆H^**≠**^ kJ/mol	∆G^**≠**^ kJ/mol	Ea-∆H^≠^ kJ/mol
BPHP	Blank	28.28	−85.36	25.75	57.24	2.53
	1.25	32.34	−106.61	29.81	62.46	2.53
	2.50	33.51	−158.03	30.98	79.06	2.53
	7.50	33.90	−88.71	31.37	58.36	2.53
	12.50	34.63	−87.41	32.10	58.70	2.53
	17.50	35.23	−86.23	32.70	58.93	2.53
	25.00	34.41	−89.50	31.88	58.11	2.53
	37.50	52.06	−33.89	49.53	59.84	2.53
TFPHP	Blank	28.28	−85.36	25.75	57.24	2.53
	1.25	31.21	−109.21	28.68	62.11	2.53
	2.50	32.49	−158.03	29.96	78.19	2.53
	7.50	34.01	−87.70	31.48	58.17	2.53
	12.50	34.03	−88.76	31.50	58.50	2.53
	17.50	33.64	−90.87	31.11	58.76	2.53
	25.00	34.55	−88.37	32.02	58.91	2.53
	37.50	48.30	−45.37	45.77	59.57	2.53

It was observed that *E*_*a*_ for the uninhibited solution is lower than that of the inhibited solution, supposing that the dissolution of CS is slow within the existence of P_ILs_ inhibitors. The inhibitor is adsorbed on the most active adsorption sites (having the lowest energy), and the corrosion process takes place predominantly on the active sites of higher energy. Inspection of the data shows that *E*_*a*_ values increase in the presence of the **BPHP** or **TFPHP** ^46, 58, 59^, and the values of **TFPHP** were smaller than **BPHP**. This indicates that the energy barrier between the reactants and the activated complex depends on the chemical composition of the inhibitor. Because of the expanded energy hindrance for metal dissolving, this information demonstrates how the inhibitors can restrict consumption. The creation of a coating layer covering the surface goes about as an energy, furthermore, mass exchange boundary, raising the activation energy.

The enthalpy *∆H*^*≠*^ and entropy *∆S*^*≠*^ of activation were frequently determined by utilizing the substitutional recipe of the Arrhenius equation, change state condition as follows:

10 $$Ln \, \left( {I_{Lim} /T} \right) \, = \, ln\left( {R/Nh} \right) \, + \, (\Delta S^{\ne} /R) (\Delta H^{\ne} /RT)$$

where *h* is Planck’s constant, *N* is Avogadro’s number, and *T* is the temperature. From Table 5, the E_a_ and *∆H*^*≠*^ values shifted similarly, allowing us to confirm the known thermodynamic response between the E_a_ and *∆H*^*≠*^ as Eq. (11), which is equivalent to the average value of *RT* (2. 53 kJ/mol) at the average temperature (308 K) of the domain investigation.

11 $$E_a - H^{\ne} = \, RT$$

*∆H*^*≠*^ positive values show that forming the activated complex is an endothermic process. *∆S*^*≠*^ values can be determined from the intercepts (equal to *ln (R/Nh)* + *(∆S*^*≠*^*/R)*). The negative *∆S*^*≠*^ values of the inhibitors indicated that the activated complex within the rate-determining step addresses association rather than dissociation. This means that increasing ordering occurs on going from reactants to activate complex, and this may result from the P_ILs_ inhibitor molecules’ adsorption from the acidic solution and may be viewed as a quasi-substitution process between the water molecules at the CS electrode and the P_ILs_ substance in the aqueous phase. The change in free energy activation (*∆G*^*≠*^) was determined from the Arrhenius with the Eq. (12):

12 $$\Delta G^{\ne} = \, \Delta H^{\ne} - TS^{\ne}$$

*∆G*^*≠*^ values are positive, increasing in the inhibited case more than in the blank case^10, 60, 61^.

### Atomic absorption spectroscopy measurements (AAS)

AAS is a sensitive, relatively affordable, spectrometric element-selective detector, making it ideal for determining a wide range of elements at trace and ultra-trace levels. Additionally, based on the solubility of the corrosion products, it is a powerful analytical approach used to forecast the corrosion rate in various media, including acidic, basic, and neutral media. Based on the amounts of iron (Fe^*2*+^) in the protected and unprotected systems, the absorbance percentage inhibition efficiency (*%€AAS*) of the P_ILs_ on the CS surface in 8M *H*_*3*_*PO*_*4*_ solution was obtained using Eq. (13)^61^. According to the AAS data in Table 6, solutions with **BPHP** inhibitor have lower concentrations of (Fe^2+^) as the temperature drops or the concentration rises than solutions without inhibitor. **BPHP** inhibitor has lower ions of (Fe^2+^) because it is more effective at inhibiting corrosion than **TFPHP**^62^. Therefore, inhibitors have a strong indication for inhibiting the corrosion of CS.

13 $$\%\, \EUR AAS = \left( {1 - \frac{{C_{inh} }}{{C_{blank} }}} \right) \times { 1}00$$

where *C*_*blank*_ and *C*_*inh*_ are (Fe^2+^) ions concentrations in the absence and presence of the P_ILs_ inhibitors.

**Table 6:** Atomic absorption spectroscopy data shows the effect of P_ILs_ with different concentrations and temperatures on iron ions and the absorbance percentage inhibition efficiency (*%€AAS*).

Samples	[Fe^2+^] (mg L^−1^)	Signal absorbance	%€AAS
Iron + 8 M H_3_PO_4_ (293 K)	109.30	0.65700	–
Iron + 8 M H_3_PO_4_ (298 K)	110.10	0.69018	–
Iron + 8 M H_3_PO_4_ (308 K)	121.50	0.85700	–
Iron + 8 M H_3_PO_4_ (318 K)	124.90	1.18630	–
Iron + 8 M H_3_PO_4_ + 37.5 × 10^–5^ M BPHP (293 K)	27.36	0.19900	74.97
Iron + 8 M H_3_PO_4_ + 37.5 × 10^–5^ M TFPHP (293 K)	33.26	0.33560	69.57
Iron + 8 M H_3_PO_4_ + 1.25 × 10^–5^ M BPHP (293 K)	87.41	0.43050	20.03
Iron + 8 M H_3_PO_4_ + 37.5 × 10^–5^ M BPHP (293 K)	27.36	0.19900	74.97
Iron + 8 M H_3_PO_4_ + 37.5 × 10^–5^ M BPHP (293 K)	27.36	0.19900	74.97
Iron + 8 M H_3_PO_4_ + 37.5 × 10^–5^ M BPHP (298 K)	53.35	0.40223	51.54
Iron + 8 M H_3_PO_4_ + 37.5 × 10^–5^ M BPHP (308 K)	73.80	0.45400	39.26
Iron + 8 M H_3_PO_4_ + 37.5 × 10^–5^ M BPHP (318 K)	89.60	0.72400	28.26

### UV–visible analysis

**Figure 8:**
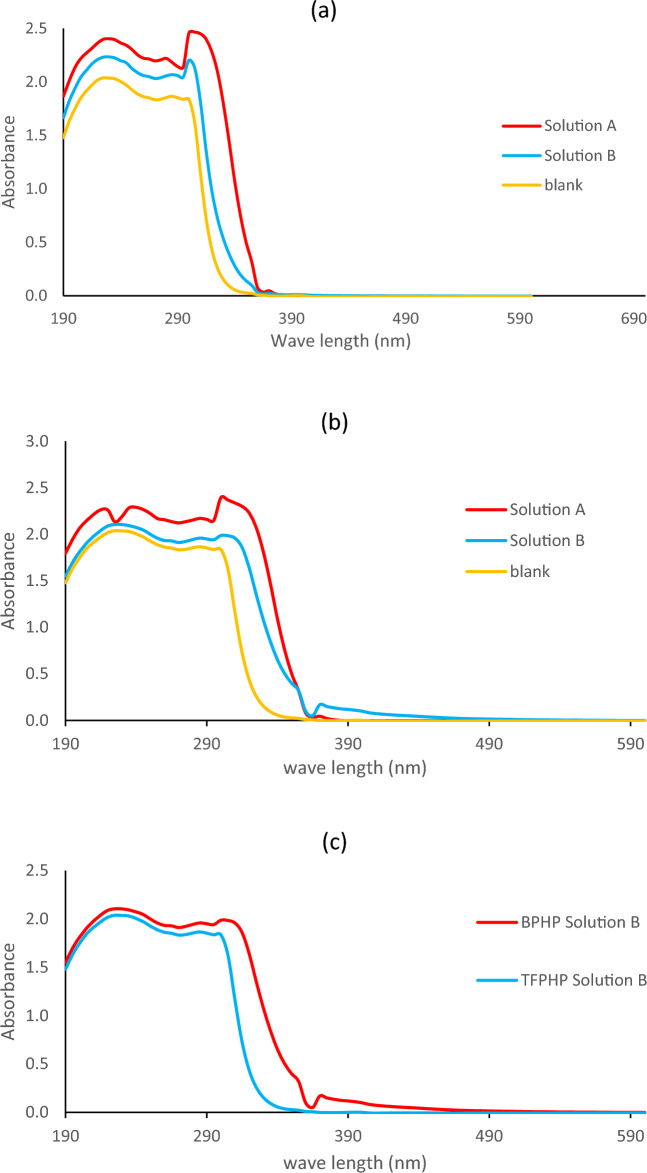
Spectra of uv–visible comparison at same concentration 37.5 × 10^–5^ M and temperature 293 K. (**a**) **TFPHP** before and after immersing in CS with blank. (**b**) **BPHP** before and after immersing in CS with blank. (**c**) **BPHP** and **TFPHP** after corrosion.

To demonstrate how a complex form, UV–VIS analysis was used with the metal cations (Fe^2+^) and P_ILs_ under study. Without adding any inhibitor, the CS electrode was subjected to the corrosive 8 M H_3_PO_4_ electrolyte at 293 K (**Blank**). The two PILs were also dissolved at a high molar concentration (37.5 × 10^–5^ M) in the same corrosive electrolyte used for the blank sample (**solution A = P**_**IL**_** + 8 M H**_**3**_**PO**_**4**_). In addition, a different solution (**solution B = P**_**IL**_** + CS + 8 M H**_**3**_**PO**_**4**_) contained a CS electrode and a predetermined molar concentration of P_ILs_ (37.5 × 10^–5^ M) dissolved and submerged once more in the same corrosive electrolyte (8 M H_3_PO_4_) at 293 K. For each P_ILs_ inhibitor, the UV was measured for the three different solutions ^11^, and the absorption wavelengths were noted and demonstrated in Fig. 8. The resulting wavelengths for the blank solution were 225, and 300 nm, which could be attributed to π–π*(*C* = *C*) and n–π* (C = O) transition, respectively. While in the case of solution A, the observed absorption values for the C=C bond due to (π–π*) were 235 nm, and the C=O bond due to (n–π*) was 310 nm for **TFPHP**. For **BPHP**, recorded high absorbance (hypochromic shift) at 240 and 320 nm is employed for C=C (π–π*) and C=O (n–π*), respectively. Additionally, for solution B, the absorption peak values were changed and shifted to 230 nm for C=C (π–π*), 305 nm for C=O (n–π*) in the case of **TFPHP**, and for **BPHP,** the absorption values appeared at 235 nm for C=C (π–π*) and for 310 nm C=O (n–π*) (bathochromic shift)^63^. Moreover, the results mentioned above reveal an important distinction in the absorption peaks in Fig. 8 between **BPHP** and **TFPHP**, suggesting that **BPHP** is the most effective inhibitor. This spectroscopic method provides proof of the complex formation between P_ILs_ inhibitors and metallic electrodes^64^.

### SEM and EDX spectroscopy

**Figure 9:**
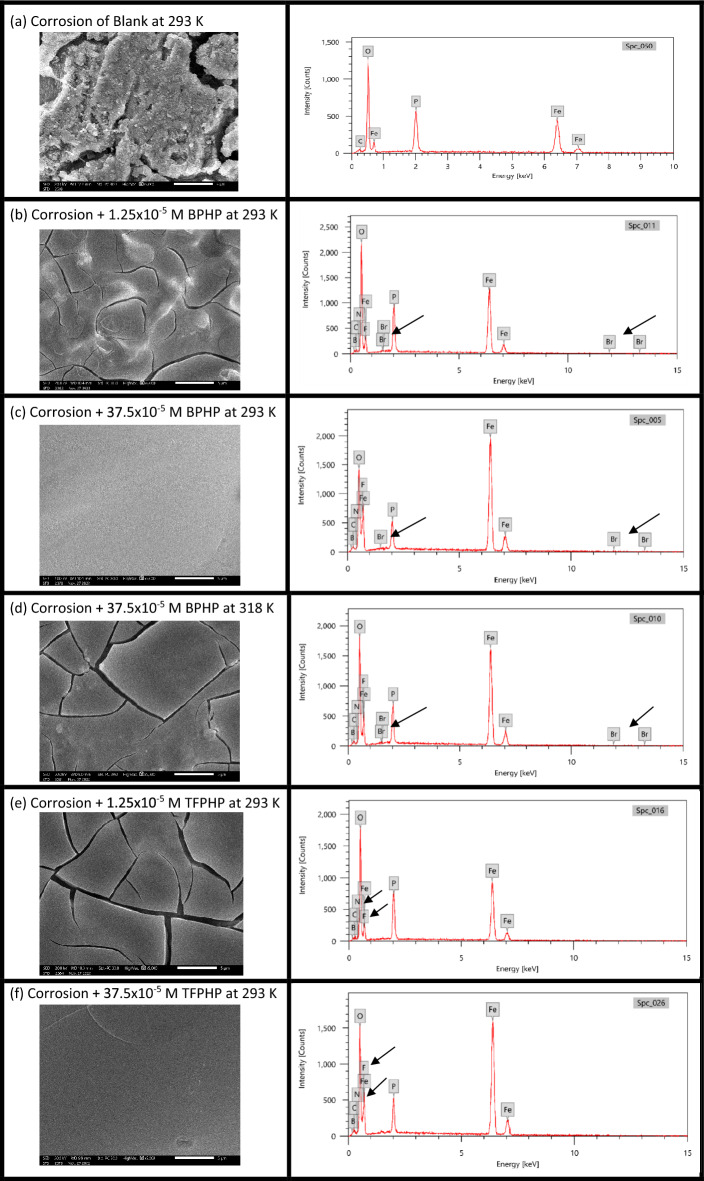
(**a**–**f**) SEM and EDX of CS surface with and without P_ILs_ at different temperatures and concentrations. (**a**) CS without P_Ils_. (**b**, **e**) CS with 1.25 × 10^–5^ M P_ILs_ at 293 K (**c**, **f**) CS with 37.5 × 10^–5^ M P_ILs_ at 293 K (**d**) CS with 37.5 × 10^–5^ M **BPHP** at 318 K.

To support the results mentioned above, films on CS surfaces were examined using an SEM–EDX study after exposure to aggressive acidic media. The distinct elements present on the CS surface of each film can be identified using the EDX technique, as can the elemental changes that resulted during immersion in 8M H_3_PO_4_ solution free of and with P_ILs_ under various concentrations and temperatures^65, 66^. The results of the EDX spectra in Fig. 9 demonstrate that the key elements of the existence of the element on the CS surface are shown by the solution-free inhibitor (blank). It is also observed in Table 7 that the percentage atomic content of *O* and *P* remarkably reduced by changing inhibitor and temperature due to CS surface coverage by a homogeneous adsorbed film. Additionally, the surface characterization of CS samples (×5000), where the surface was highly destroyed under inhibitor-free conditions, confirmed the adsorption of P_ILs_. After the adsorption of P_ILs_, By the way, the hetero atoms such as *C* and *O* indicate that at the active sites of adsorption, the surface becomes smoother when the percent of *Fe* and *C* increase, *O* and *P* decrease with increasing inhibition of the corrosion. The inhibition and the surface smoothing, as observed from SEM Fig. 9, are directly probational with increasing the concentration; the least smoothing surface is blank (%*Fe* = 37.32, %*C* = 1.21, %*O* = 54.81 and %*P* = 15.66) and for 1.25 × 10^–5^ M (%*Fe* = 47.29, %*C* = 3.51, %*O* = 33.13 and %*P* = 8.93) is less smooth than 3.75 × 10^–4^ M (%*Fe* = 62.66, %*C* = 3.34, %*O* = 21.55 and %*P* = 5.29) at same temperature 293 K for **BPHP** and the same competition for **TFPHP**. As the temperature increases at 37.5 × 10^–5^ M, the inhibition decreases at 318 K (%*Fe* = 52.28), more than 293 K (%*Fe* = 62.66) for **BPHP**.

**Table 7:** Percentage of elemental analysis content derived from EDX spectra of P_ILs_.

No	Samples	Fe	C	O	P
a	Blank (293 K)	37.32	1.21	54.81	15.66
b	Corrosion + BPHP 1.25 × 10^–5^ M (293 K)	47.29	3.51	33.13	8.93
e	Corrosion + TFPHP 1.25 × 10^–5^ M (293 K)	39.44	3.37	36.97	10.26
c	Corrosion + BPHP 37.5 × 10^–5^ M (293 K)	62.66	3.34	21.55	5.29
f	Corrosion + TFPHP 37.5 × 10^–5^ M (293 K)	56.71	2.52	25.78	6.01
c	Corrosion + BPHP 37.5 × 10^–5^ M (293 K)	62.66	3.34	21.55	5.29
d	Corrosion + BPHP 37.5 × 10^–5^ M (318 K)	52.28	3.18	28.15	6.89

On the other hand, **BPHP** has more inhibition than **TFPHP** at the same concentration and temperature because **BPHP** forms more surface coating than **TFPHP**. However, it can be noticed from EDX recorded in the presence of P_ILs_ in Fig. 9 the appearance of new peaks related to the *N* atom, which act as active centers of these inhibitors for adsorption and forming a protected film on the CS surface. On the other hand, Br appeared on the EDX diagram for **BPHP** due to the difference between two structures: the terminal group, *Br*, for **BPHP** and *CF*_*3*_ group for **TFPHP** and the group *BF*_*4*_^-^ the negative ion for both inhibitors where *F* appeared in the EDX.

### Atomic force microscopy (AFM)

**Figure 10:**
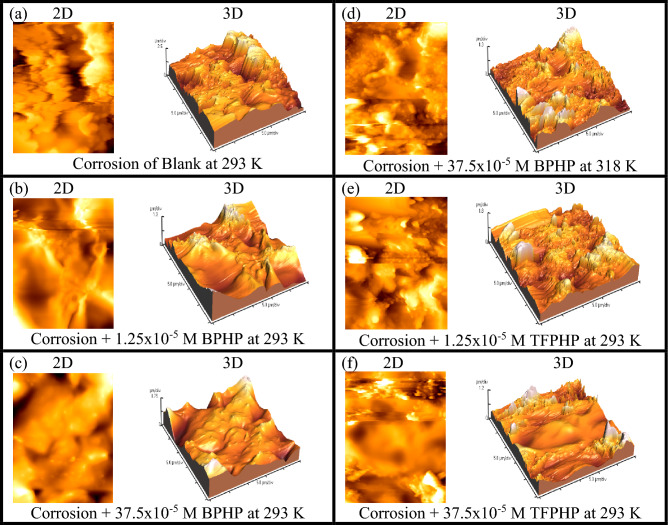
(**a**–**f**) AFM graphs of blank and P_ILs_ with different concentrations and temperatures. (**a**) CS without P_Ils_. (**b**, **e**) CS with 1.25 × 10^–5^ M P_ILs_ at 293 K. (**c**, **f**) CS with 37.5 × 10^–5^ M P_ILs_ at 293 K (**d**) CS with 37.5 × 10^–5^ M **BPHP** at 318 K.

Presently, several imaging techniques are now available that can provide accurate three-dimensional topographies and information about the irregularities on the sample surface. One uses an AFM to provide quantitative analysis rather than SEM micrographs' qualitative analysis^67^. Figure 10a–f illustrates the 2D and 3D morphological characteristics of CS surfaces immersed in 8 M H_3_PO_4_ at various temperatures and concentrations with and without P_ILs_ inhibitor **BPHP** or **TFPHP**. Table 8 lists the values for the average surface roughness (*Ra*), which reflects the deviation in height, the root means square roughness (*Rq*), which represents the deviation in surface heights, and the maximum peak to valley depth (*Rp-v*).

**Table 8:** Atomic force microscopic parameters for CS in 8 M H_3_PO_4_ with and without P_ILs_ at different concentrations and temperatures.

No	Samples	Ra (μm)	Rq (μm)	Rp-v (μm)	% I_Eff_
a	Blank (293 K)	0.62	0.79	5.00	0.00
b	Corrosion + BPHP 1.25 × 10^–5^ M (293 K)	0.30	0.38	2.55	20.63
e	Corrosion + TFPHP 1.25 × 10^–5^ M (293 K)	0.44	0.56	3.68	15.87
c	Corrosion + BPHP 37.5 × 10^–5^ M (293 K)	0.16	0.20	1.50	76.19
f	Corrosion + TFPHP 37.5 × 10^–5^ M (293 K)	0.18	0.25	2.31	71.43
c	Corrosion + BPHP 37.5 × 10^–5^ M (293 K)	0.16	0.20	1.50	76.19
d	Corrosion + BPHP 37.5 × 10^–5^ M (318 K)	0.27	0.35	2.59	49.37

**Figure 11:**
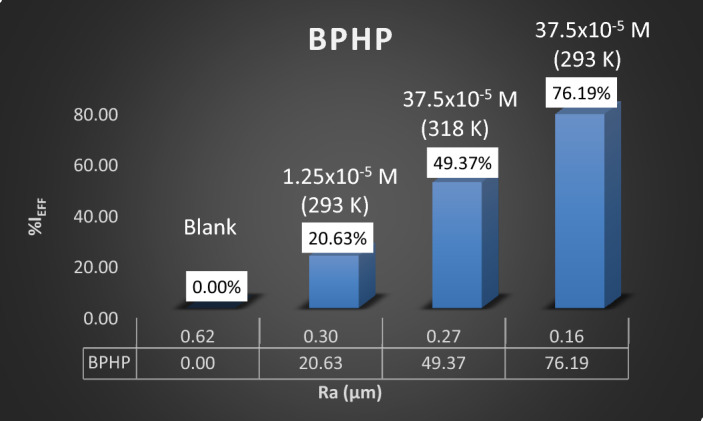
Relation between average roughness (*Ra*) and inhibition efficiency (%${I}_{Eff}$) at different concentrations and temperatures for **BPHP** and blank.

The P_ILs_ layer on the CS surface in solution-free inhibitors is shown in Fig. 10. Direct contact with 8M *H*_*3*_*PO*_*4*_ media totally damages the corroded CS surface with an average roughness (*Ra*) of 0.62 μm, *Rq* of 0.79 μm, and *Rp-v* of 5.00 μm. On the other hand, as the inhibition increased, the parameters roughness decreased^68^, resulting in an improvement of the surface quality film and the formation of a more uniform film that was observed by adding the optimum concentration of **TFPHP** (37.5 × 10^–5^ M) at 293 K. These parameters roughness magnitude decreased to *Ra* of 0.18 μm, *Rq* of 0.25 μm, and *Rp-v* of 2.31 μm. This coating is a barrier between the metal and the corrosive medium and considerably prevents CS deterioration. However, the surface roughness parameters decreased in the presence of **BPHP** Fig. 11, owing to the more protective activity of adsorbed inhibitor molecules on the CS surface rather than **TFPHP**, as evidenced by a decrease in average surface roughness values. Table 8 shows the percent inhibition (*%I*_*Eff*_) increased with increasing the concentration and decreasing the temperature for **BPHP**. By comparing the parameters, the values of **BPHP** were less than **TFPHP** values, which proved that **BPHP** has higher inhibition efficiency and is more adsorbed than **TFPHP**.

### UV spectral reflectance studies (Gloss value)

**Figure 12:**
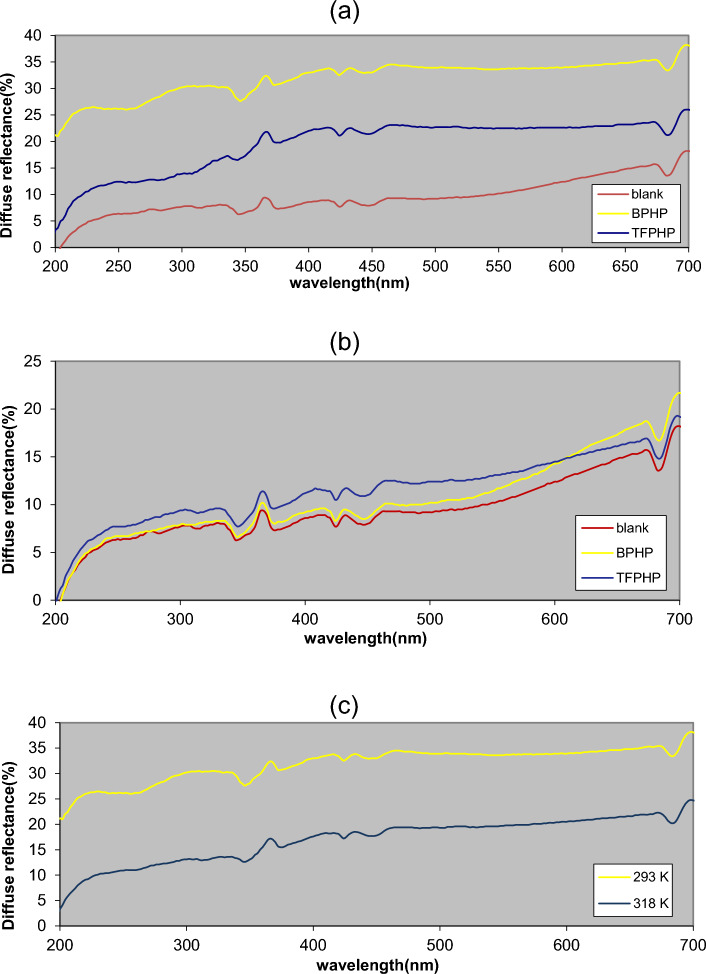
The gloss reflectance of P_ILs_. (**a**) comparison between blank and P_ILs_ with 37.5 × 10^–5^ M P_ILs_ at 293 K. (**b**) comparison between blank and P_ILs_ at with 1.25 × 10^–5^ M P_ILs_ at 293 K. (**c**) comparison for **BPHP** with 37.5 × 10^–5^ M at different temperatures 293 and 318 K.

UV–visible diffuse reflectance spectroscopy of the CS surface was analyzed before and after immersion in 8M H_3_PO_4_ in the 200–700 nm range. The reflectance curves examined the function spectra of the coated film on the CS surface with different P_ILs_ inhibitor concentrations at different temperatures to identify iron phosphate. The blank sample (before corrosion) showed a low gloss value (*G* = 18.2, dull), as shown in Fig. 12. Conversely, all samples coated with P_ILs_ in different conditions were electrolytically corroded, where they showed higher gloss values ^69^. In the case of the lower concentrations, the gloss value will be *G* = 21.7 (**BPHP**) and *G* = 19.2 (**TFPHP**), and optimum concentration *G* = 38.1 (**BPHP**) and *G* = 26 (**TFPHP**). Under different temperatures for **BPHP**, surface brightness was observed to increase by decreasing temperature to *G* = 38.1 at 293 K and *G* = 24.7 at 318 K. This indicates the reduction of the surface roughness and increasing the reflectance value as **BPHP** has more efficiency in inhibition rather than **TFPHP** due to forming a protective film on the metal surface.

### XPS analysis

X-ray photoelectron spectroscopy (XPS) was utilized to analyze the elemental composition and chemical bonds which formed on the CS surface before and after the adsorption of P_ILs_. This analytical technique played a crucial role in enhancing our comprehension of the underlying mechanism of adsorption.

**Figure 13:**
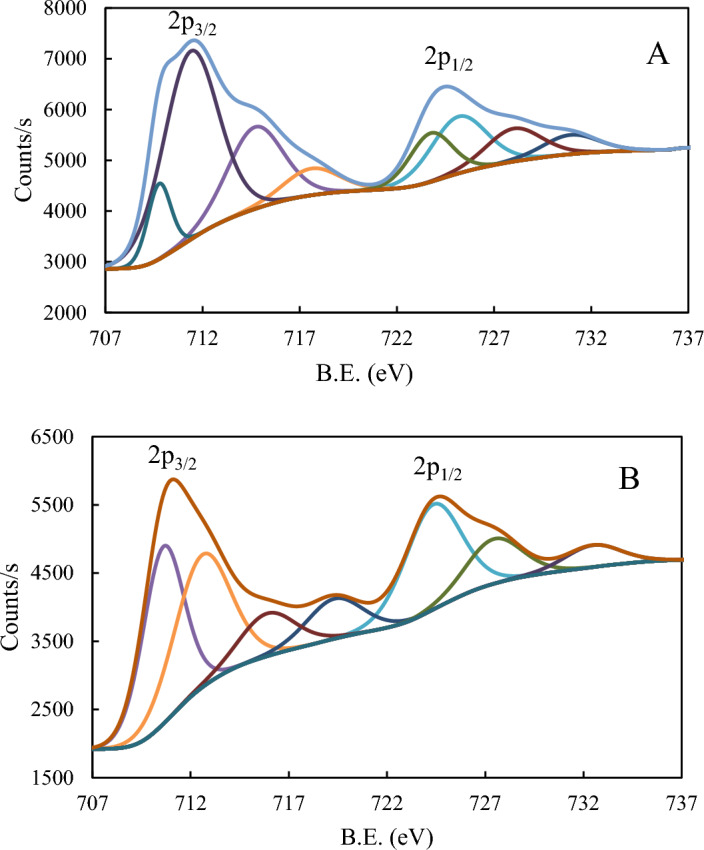
XPS scan of Iron (**A**) and after (**B**) adsorption.

XPS of iron, Fig. 13, showed two main peaks corresponding to 2p_3/2_ appeared at 711.48 (711.00) and 2p_1/2_ at 724.48(724.48) eV with a difference of 13 (13.48) eV, before and (after)adsorption indicating the presence of iron as various iron substrate Fe_3_O_4_ or FeO (Fe^2+^) and Fe_2_O_3_ (Fe^3+^)^70, 71^. However, The binding energy values in the 709–714 eV range confirm the presence of Fe (III) and/or Fe (II) and/or the formation of Fe–O–C bond^72^. Table 9 summarizes all peaks corresponding to Fe-binding energies before and after adsorption, showing that Different environments surround Fe-ions. The peaks at 712.59 and 727.43 eV with ΔE = 14.84 eV may be attributed to the presence of Fe_3_O_4_. The appearance of a satellite of 719.32 eV and 715.89 eV (ΔE = 3.43 eV) may correspond to bonding with fluoride ion^73^.

**Table 9:** XPS binding energies and separation (ΔE) for iron before and after adsorption.

Blanck			After adsorption		
2p_3/2_	2p_1/2_	ΔE	2p_3/2_	2p_1/2_	ΔE
709.77	723.74	13.97	710.60	724.34	13.74
711.38	725.18	13.80	712.59	727.43	14.84
717.61	730.94	13.33	719.32	732.48	13.16
714.70	728.02	13.32	715.89	-	-

**Figure 14:**
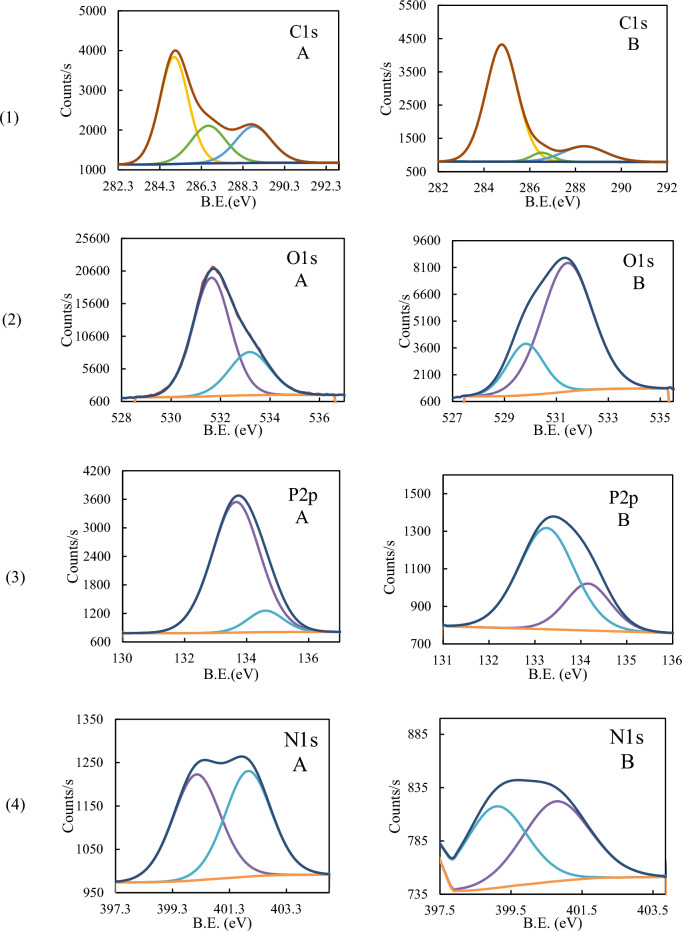

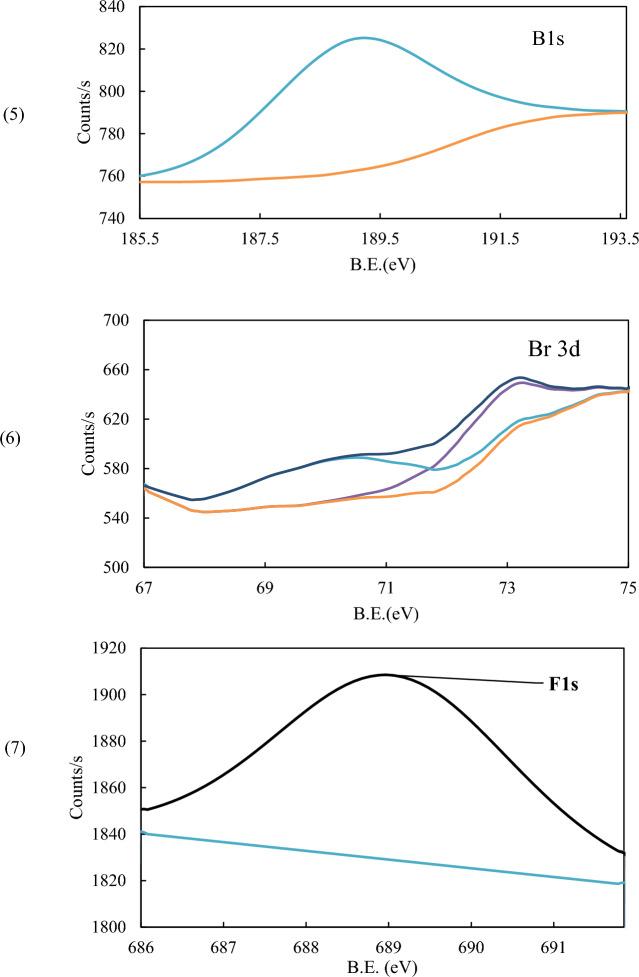
XPS scan of (1) C1s, (2) O1s, (3) P2p, and (4) N1s, before (**A**) and after (**B**) adsorption of **BPHP** and (5) B1s, (6) F1s, and (7) Br 3d after adsorption process.

Figure 14 shows the XPS spectra for C1s, O1s, P2p, and N1s, before and after adsorption. Three peaks of C1s at 284.99, 286.64, and 288.81 eV in the blank sample showed a slight shift in the B.E. values to 284.78, 286.53, and 288.38eV after treatment by inhibitor (Fig. 141-A, B). These peaks correspond to aromatic C, CN, and CO^72, 74^. A very large decrease in the peak area of peaks corresponding to CN or CO indicates the change in their media. For O1s spectrum was deconvoluted into two peaks at 531.64 and 533.18 eV corresponding to the Fe–O and O-C bonds, respectively, whereas the peaks were shifted to lower binding energies after adsorption at 531.58 and 529.97 eV, which could be attributed to its bonding to O-C, O-Fe(II)and/or Fe(III) ions (Fig. 142-A, B)^72^. P2p spectrum showed two deconvoluted peaks at 133.69 and 134.66 eV, which may attributed to PO_3_ P and PO_4_P, respectively, showing a small shift after adsorption to 133.25 and 134.16, with a notable change in the peak area, Fig. 14(3-A, B)^75^. On the other hand, N1s showed a relative decrease in the peak intensity and peak areas that appeared at (400.15, 401.96) eV before adsorption and (399.08, 400.65) eV after adsorption. The binding energies could be attributed to N = C/N^+^–C and N–H before that could have disappeared after adsorption (Fig. 14(4-A, B)^76^.

The XPS scan after adsorption showed the presence of Boron (B), Fluorine (F), and Bromine (Br), which are shown in detail in Fig. 14(5–7). B1s displayed a characteristic boride peak at 189.1 eV, which is a smaller value than that of B-B that could be attributed to metal or carbon center attachment^77, 78^. This is also can be evidenced by the difference in the N1s and C1s scans before and after adsorption. Fluorine showed F1s peak at 689.2 eV, which may be attributed to F–C bonding confirming its existence^79^. Bromine displayed two peaks corresponding to Br 3d with binding energies (B.E.) of 72.77 and 70.20 eV (ΔE = 2.57), which may be attributed to Br–C and/ charge transfer from Br to iron in either oxidation state II and/or III. The presence of B, F and Be elements confirms the adsorption of **BPHP** as a protective inhibitor on the CS surface^80^.

### Computational investigation study

Quantum chemical descriptors, including *E*_*HOMO*_*, E*_*LUMO*_, Energy gap *(∆E* = *E*_*LUMO*_*—E*_*HOMO*_), chemical hardness, chemical softness, electronegativity, chemical potential, proton affinity, electrophilicity, and nucleophilicity, are well-known for being beneficial and efficient tools in investigations of metal corrosion. Sup.Tables (1–4) provide an overview of the selected P_ILs_ atoms of global molecular properties in the gas phase and aqueous solution^81, 82^. The following will cover the relationships between descriptors and the order of corrosion inhibition efficiencies.

**Figure 15:**
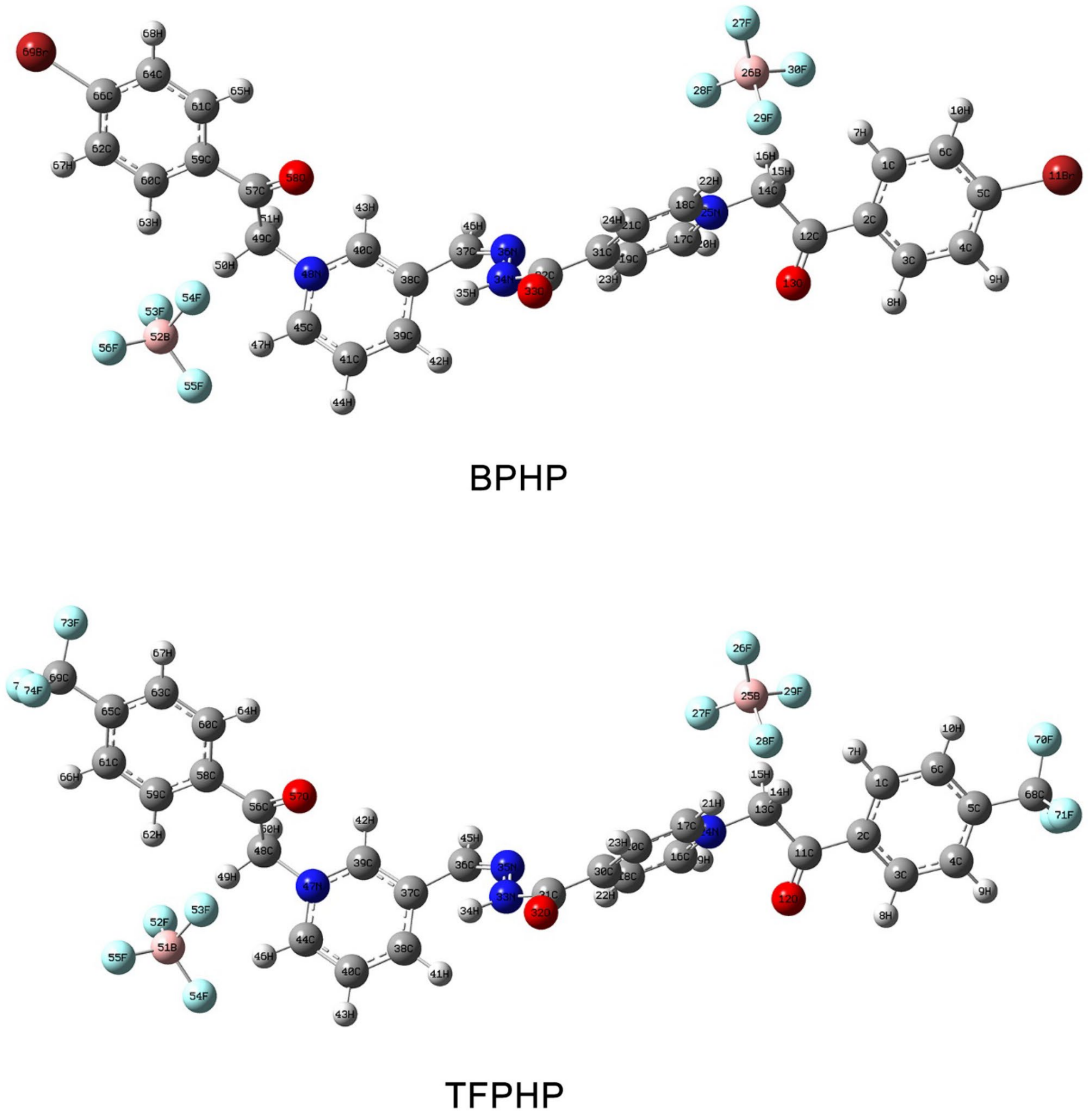
Optimized structures configuration geometry of P_ILs_.

The Figured quantum compound properties for **BPHP** & **TFPHP** in the gas and fluid phases are given in Table 10. The geometry optimization is displayed in Fig. 15. The capacity of a particle to adsorb onto the metal surface is related to the hypothesis of frontier molecular orbital (*FMO*). *E*_*HOMO*_ alludes to the capacity of atoms to give electrons to the iron surface with empty "*d*" orbitals. The higher the energy level of the HOMO (*E*_*HOMO*_), the more susceptible the ligand is to donate the electrons to the iron atoms to form a stronger bond. This could illustrate the compound's effectiveness in inhibiting the atoms' capacity to give electrons.

**Table 10:** Data of calculated quantum parameters for P_ILs_ in gas and aqueous phase.

State	Gas		Aqueous	
P_ILs_	BPHP	TFPHP	BPHP	TFPHP
E_HOMO_ (eV)	−7.3837	−7.9947	−7.2513	−7.5212
E_LUMO_ (eV)	−4.0686	−4.1582	−3.1932	−3.2128
∆E_gap_ (eV)	3.3151	3.8365	4.0581	4.3084
I (eV)	7.3837	7.9947	7.2513	7.5212
A (eV)	4.0686	4.1582	3.1932	3.2128
X (eV)	5.7262	6.0765	5.2223	5.3670
P_i_ (eV)	−5.7262	−6.0765	−5.2223	−5.3670
µ_d_ (Debye)	11.8266	12.5062	17.5000	16.6904
ƞ (eV)	1.6576	1.9183	2.0291	2.1542
σ (eV^−1^)	0.6033	0.5213	0.4928	0.4642
ω (eV)	9.8907	9.6242	6.7204	6.6857
∆E_b.d_ (eV)	−0.4144	−0.4796	−0.5073	−0.5386
∆N (eV)	0.3843	0.2407	0.4381	0.3790
T.E (Hartee)	−7522.6624	−3049.8514	−7522.6438	−3049.8387
∆E_steel/inh_ (eV)	0.2447	0.1112	0.3894	0.3095
ω^-^ (eV)	12.9610	12.9022	9.5851	9.6385
ω^+^ (eV)	7.2349	6.8258	4.3629	4.2715
∆ω^±^ (eV)	7.1577	6.7483	4.2585	4.1677

**Figure 16:**
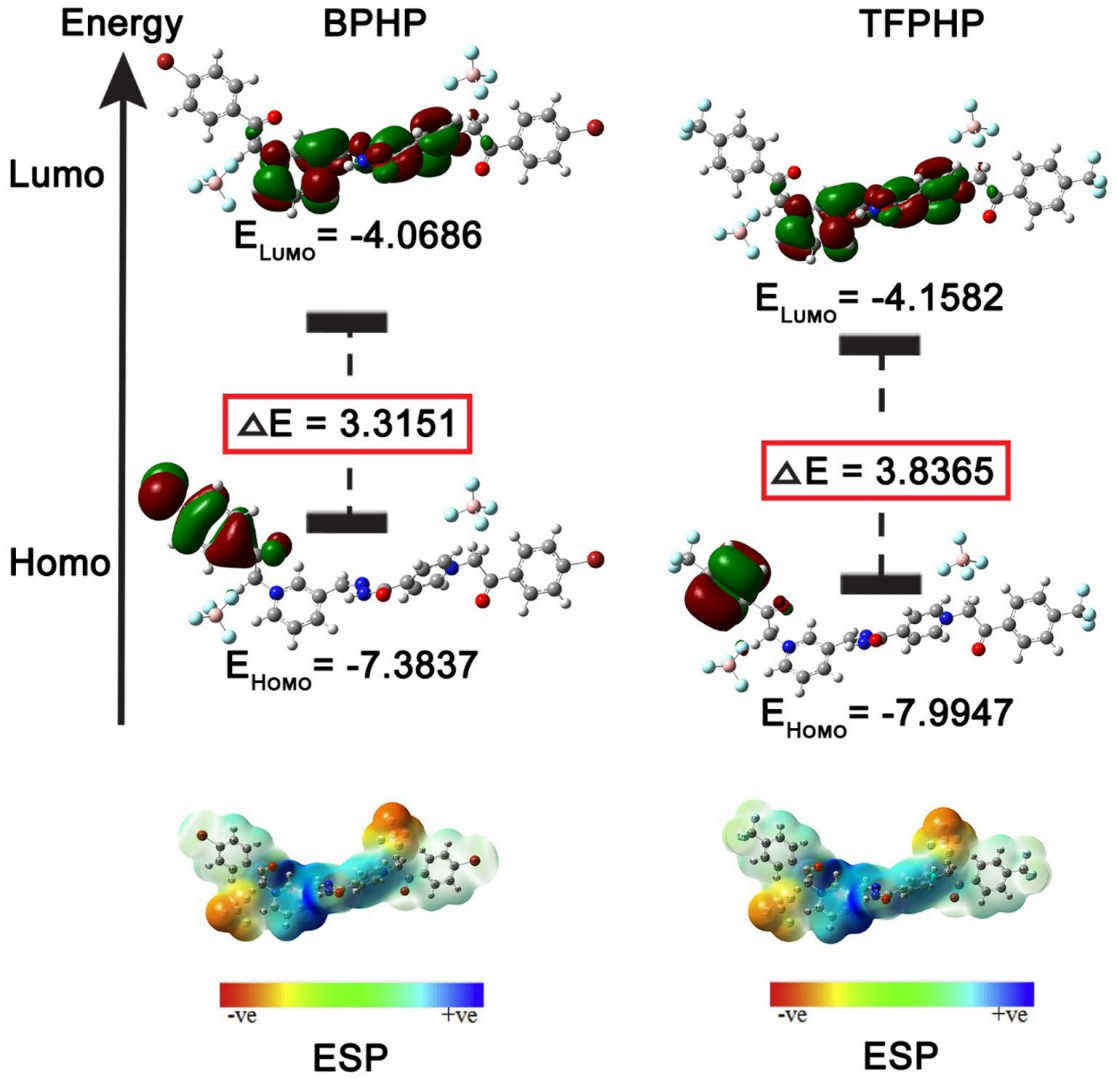
Frontier molecular orbital for HOMO–LUMO distribution energy and molecular electrostatic potential of P_ILs_.

For this reason, **BPHP** is more effective than **TFPHP** in electron donation in the two phases. In concurrence with the calculation’s outcomes, the *E*_*HOMO*_ of **BPHP** is the biggest. Subsequently, this compound is viewed as more reasonable for adsorption on the metallic surface through the pyridine ring, which is a rich source of electrons. In contrast, *E*_*LUMO*_ demonstrates the acceptance of electrons by the atoms where the lower value of *E*_*LUMO*_, the more prominent the inhibitor effectiveness. The energy gap *(∆E*_*gap*_) plays a vital role in the reactivity of particles toward the metal surface. The lower value of *∆E*_*gap*_ is the more tendency of atoms to adsorb on the metal surface. Table 10 shows that *∆E*_*gap*_ for **TFPHP** in the gas phase is lower than in water. On the other hand, *∆E*_*gap*_ values for **BPHP** are lower than those for **TFPHP**, showing that **BPHP** has a high reactivity in both phases. The calculations of (*FMOs*) of (**BPHP** & **TFPHP**) were performed by *B3LYP/6-311g (d,p)* Gaussian-09 program using density functional theory (DFT)^83, 84^, and the Gaussian View 5.0 was used to show the structural forms in Fig. 16.

The other parameters like hardness ($\eta$), softness ($\sigma$), electron back donation (${\Delta E}_{b.d}$), work function ($\Delta N$) and electronegativity ($X$) are calculated from the relations:

14 $$I \, = E_{HOMO} \quad A \, = E_{LUMO} \quad P_i = X$$

15 $$X = \frac{{\left( {I + A} \right) }}{2}\quad \eta \, = \frac{{\left( {I - A} \right) }}{2}\quad \sigma \, = \frac{1}{\eta }\quad \Delta E_{b.d} = \frac{ - \eta }{4}$$

16 $$\omega \, = \frac{P_i^2 }{{2\eta }}\quad \omega^- = \frac{{\left( {3I + A} \right)^2 }}{{16\left( {I - A} \right)}}\quad \omega^+ = \frac{{\left( {I + 3A} \right)^2 }}{{16\left( {I - A} \right)}} \quad \Delta \omega^\pm = \, \left( {\omega^+ - \frac{1}{\omega^- }} \right)$$

17 $$\Delta N \, = \frac{{\left( {X_{Fe} -- X} \right)}}{{2\left( {\eta_{Fe} + \eta } \right)}}\quad \Delta E_{steel/P_{ILs} } = \frac{{\left( {X_{Fe} -- X_{inh} } \right)^2 }}{{4\left( {\eta_{Fe} + \eta_{inh} } \right)}}$$

where (*I*) ionization potential, (*P*_*i*_) chemical potential, (*A*) electron affinity, (*X*_*Fe*_) electronegativity of *Fe* = 7 eV, ${\eta}_{Fe}$ The hardness of *Fe* = 0^85^, $\Delta N$ is positive, meaning electrons transfer to the *Fe* surface from the inhibitors, and *∆E*_*steel/inh*_ shows the interaction between **BPHP** or **TFPHP** and metal surface. The inhibitor **BPHP** has high work function, high softness, a less negative value of the back donation, high negative total energy (*T.E*), low hardness, high *∆E*_*steel/*PILs_ and low electronegativity **BPHP** is more inhibition efficiency than **TFPHP** in the two phases.

The descriptors *ω*^+^*, ω*^*-*^*,* and *∆ω*^±^ mean electron accepting, electron-donating and net electrophilicity, respectively, where they increased as inhibition efficiency increased with decreasing energy gap^86^. On the other hand, dipole moment (*µ*_*d*_) measures the polarity of the chemical bond in the molecules. In the aqueous phase, µ_d_ has a larger value for **BPHP** than **TFPHP**, so **BPHP** is more anticorrosive, which matches experimental data. It is not a condition that all parameters agree with the practical information, but the majority agree with these results.

### Molecular electrostatic potential

The inhibitors' absorption on the metal surface is shown by the molecular electrostatic potential (MEP). In Fig. 16, the atoms of *O, N* and some of the *C* atoms for **BPHP** & **TFPHP** matrices carry negative charges. The highly negatively charged atoms can exchange electrons with the positive sites on the surface of the metal^87^, Supplementary Tables 1–4 shows the distribution of the atomic charges of Mulliken on the atoms of **BPHP** & **TFPHP**. The best atoms in gas and aqueous phases are (*C5*, *O13*, *N25*, *O33*, *N34*, *C38*, *N48*, *O58*) for **BPHP** and (*C5*, *O12*, *N24*, *O32*, *N33*, *C37*, *N47*, *O57*) for **TFPHP**. So that atoms are responsible for a nucleophilic attack on the surface of the CS. Figure 16 shows the molecular electrostatic potential and electron density (*ED*) regions. The high *ED* is shown in red color, and the low *ED* is a blue color. *ED* decreases in the following order: red > orange > yellow > green > blue ^84^. The high *ED* (yellow to red color) is localized on *O* and *N* atoms for **BPHP** & **TFPHP**. The low *ED* (green to blue color), on the other hand, is located on a few carbon atoms.

### Fukui indices and local dual descriptors

**Figure 17:**
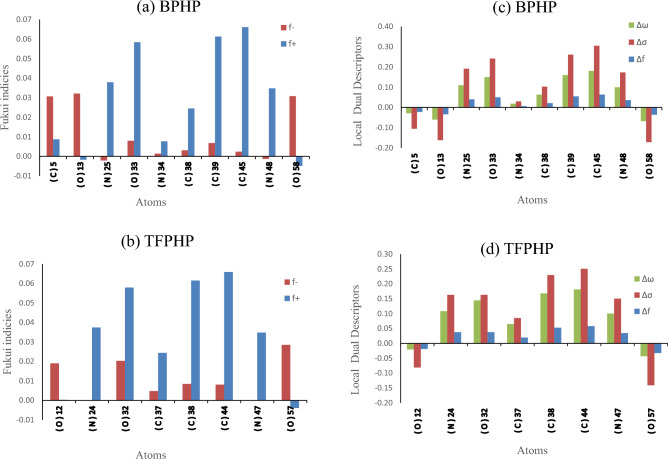
Fukui indices (**a**, **b**) and local dual descriptors (**c**, **d**) of different atoms with respect to Mulliken charges of P_ILs_.

Fukui indices and Local Dual Descriptors describe the electrophilic and nucleophilic attack in the gas phase. The calculated condensed Fukui functions using Mulliken charges (*f*_*k*_^+^, *f*_*k*_^*−*^), local electrophilicity (*ω*_*k*_^+^, *ω*_*k*_^*−*^) and local softness (*σ*_*k*_^+^, *σ*_*k*_^*−*^) are reported in Sup.Tables (1,2). To facilitate the comparison between the possible sites for nucleophilic and electrophilic attacks on any atom k, we have calculated (*Δf*_*k*_, *∆σ* and *∆ω*), which corresponds to the difference between (*f*_*k*_^+^, *f*_*k*_^*−*^), (*σ*_*k*_^+^, *σ*_*k*_^*−*^) and (*ω*_*k*_^+^, *ω*_*k*_^*−*^), respectively ^10, 88^. When comparing the atoms to determine the most reactive site consequently, the positive values of *∆f*, *∆σ*, *∆ω*, i.e., > 0, would be most favorable for a nucleophilic attack like (*N25*, *O33*, *N34*, *C38*, *C39*, *C45*, *N48*) atoms for **BPHP** & (*N24*, *O32*, *C37*, *C38*, *C44*, *N47*) atoms for **TFPHP**, on the other hand, if *∆f*, *∆σ*, *∆ω* > 0, it would be favoured for an electrophilic attack like (*C5*, *O13*, *O58*) atoms for **BPHP** & (*O12* and *O57*) atoms for **TFPHP** and all are illustrated and described in the following equations and Fig. 17.

18 $$\begin{aligned} f_k^+ & = \, \rho_k \left( {N + 1} \right) \rho_k \left( N \right) \;\left( {{\text{nucleophilic}}\;{\text{attack}}} \right) \\ f_k^- & = \, \rho_k \left( N \right) \rho_k \left( {N - 1} \right) \; \left( {{\text{electrophilic}}\;{\text{attack}}} \right) \\ \end{aligned}$$

19 $$\begin{aligned} \Delta f \, & = \, \left( {f_k^+ } \right)-- (f_k^- ) \\ \Delta \sigma \, & = \, \left( {\sigma_k^+ } \right)-- \left( {\sigma_k^- } \right) \\ \Delta \omega & \, = \, (\omega_{\text{k}}^+ )-- \left( {\omega_k^- } \right) \\ \end{aligned}$$

where the electronic density site *ρ* with the number of electrons (*N*) at site *k* in a molecule.

#### Inhibition mechanism

**Figure 18:**
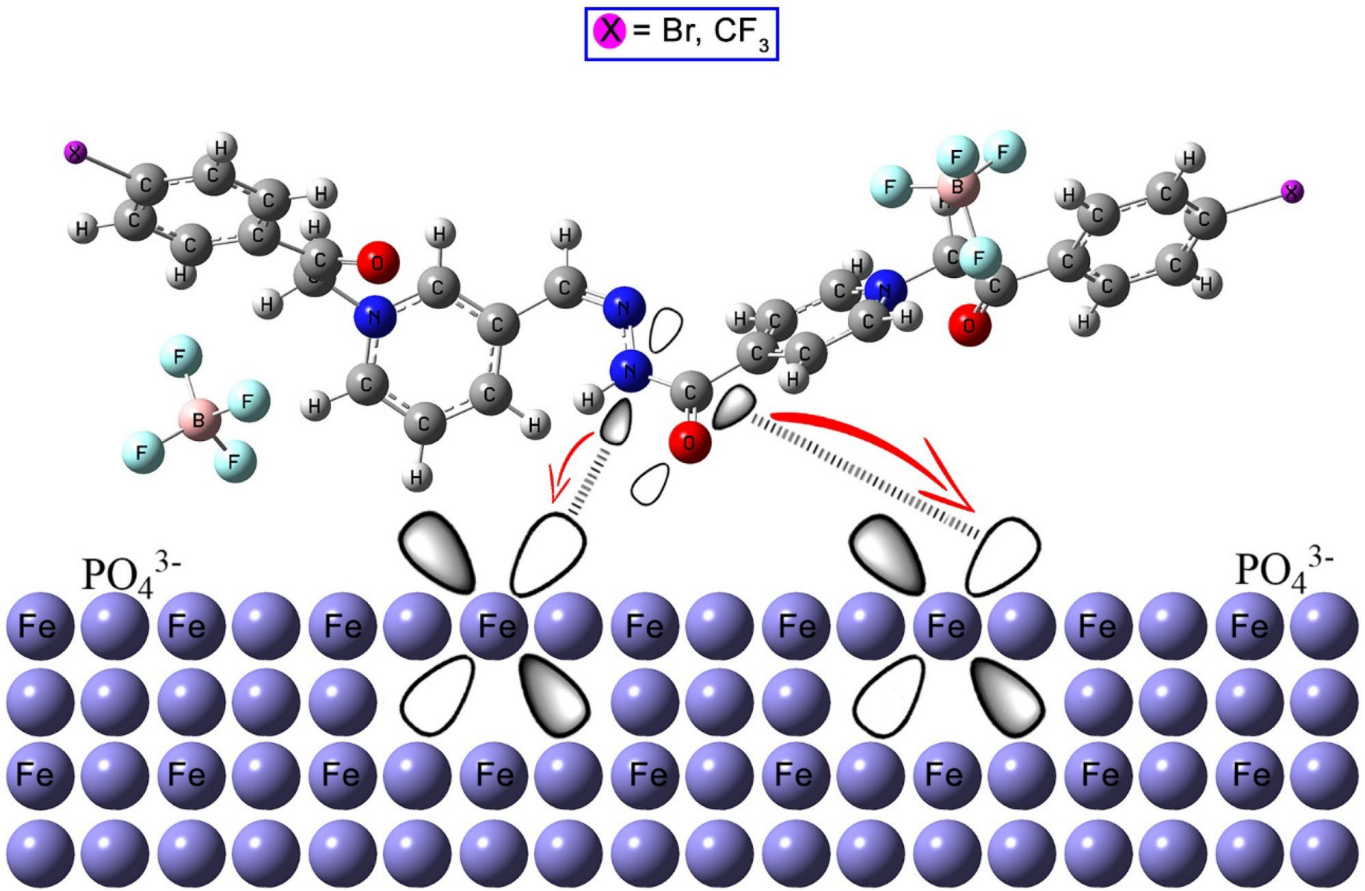
Schematic mechanism diagram of adsorption P_ILs_ on CS surface in 8M *H*_*3*_*PO*_*4*_.

According to the previous discussion, **BPHP** and **TFPHP** operate physically, chemically, or through both (physicochemical) adsorption to reduce the effectiveness of the employed solution (8 M *H*_*3*_*PO*_*4*_) on the CS surface. On the CS surface or due to the protonation of the inhibitor's heteroatoms in the bulk solution, charged species are present in the physical model. On the other hand, the chemisorption could be increased by transferring charge from the electron-rich centers (the lone pair of the heteroatom or the π-electrons of the unsaturated centers) of the inhibitor to the CS vacant d-orbitals (Fe^2+^)^89^, which reduces surface erosion as shown in Fig. 18. By comparing the *N* (−0.3014) and *O* (−0.2952) of **TFPHP**, the calculated Milliken atomic charges of *N* (−0.3020) and *O* (−0.2968) of **BPHP** are more negative. Because the CF_3_ polar group is more electron withdrawing than *Br*, the presence of the *CF*_*3*_ may reduce the amount of charge transfer from the inhibitors to the CS surface. These findings might demonstrate how **BPHP** inhibits more effectively than **TFPHP**.

## Conclusion

Finally, we found two new synthetic P_ILs_ with high efficiency of inhibition for C-steel surface and nontoxic where at different temperatures and concentrations, especially at 37.5 × 10^–5^ M with 293 K; **BPHP** and **TFPHP** were good inhibitors with inhibition percent 76.19% and 71.43% respectively. The electrochemical study of corrosion used to particular the anticorrosive new compounds where the best model of adsorption isotherm for them is the El-Awady model was applied. Temperature increase leads to decreased inhibition efficiency, resulting in the effective activation energy value variation. Furthermore, surface studies like scanning electron microscopy-energy dispersive spectroscopy (SEM–EDS) revealed the protecting capability of the investigated inhibitors. An ultra-violet visible (UV–vis.) spectroscopy study confirms the formation of the Fe^2+^–P_ILs_ complex. X-ray Photoelectron Spectroscopy (XPS) was conducted to study the formation of corrosion products and protective film over the mild steel surface. The theoretical study of DFT confirmed the experimental data and the mechanism, which showed the adsorption as physicochemical. The different characterizations showed that **BPHP** is better than **TFPHP** inhibitor due to the terminal group of **BPHP** being *Br* and *CF*_*3*_ for **TFPHP**, where *Br* is more electron rich, softer, with positive Fukui parameters than *CF*_*3*_. We hope to use the new P_ILs_ in another process, like Electroplating, to transfer layers of different metals to another metal surface.

### Supplementary Information

Supplementary Information.

## Data Availability

The data used and analyzed during the current study are available from the corresponding authors upon reasonable request.
