# Supplementary material for: Anticorrosive performance of newly synthesized dipyridine based ionic liquids by experimental and theoretical approaches
Source: Sci Rep. 2023 Nov 6;13:19197. doi: 10.1038/s41598-023-45822-9 (PMC10628253; doi:10.1038/s41598-023-45822-9)
Supplement: Supplementary file 1 — Supplementary Information. [file 41598_2023_45822_MOESM1_ESM.docx]

**Anticorrosive performance of newly synthesized dipyridine based ionic liquids by experimental and theoretical approaches.**

*Amira H. E. Moustafa ^1^, Hanaa H. Abdel-Rahman ^1^, Mohamed Hagar ^1^, Mohamed R. Aouad ^2^, Nadjet Rezki ^2^, Sherif A. A. Bishr ^1^*

1. **Synthesis of the dipyridines ionic liquids**

- ***Synthesis and characterization of 1-(2-(4-brimophenyl)-2-oxoethyl)-3-((2-(1-(2-(4-subistitutedphenyl)-2-oxoethyl)pyridin-1-ium-4-carbonyl)hydrazono) methyl)pyridin-1-ium bromide*, 3(a,b)**

4-substituted phenacyl bromide (2.2 mmol) was added to a mixture of N'-(pyridin-3-ylmethylene)isonicotinohydrazide **(1)** (1 mmol) in acetonitrile (30 mL). The reaction mixture was then refluxed for 6 hours. Under reduced pressure, the solvent was evaporated, and the precipitate produced was recovered and recrystallized from ethanol to get the necessary dicationic liquids **3 (a,b).**

- ***1-(2-(4-bromophenyl)-2-oxoethyl)-3-((2-(1-(2-(4-bromophenyl)-2-oxoethyl)pyridin-1-ium-4-carbonyl)hydrazono) methyl)pyridin-1-ium bromide*, 3(a)**

Yield: 89 %. ^1^H NMR (400 MHz, DMSO-*d*_6_): δ_H_ = 6.78 (d, 4H, *J* = 8 Hz, 2×NC**H**_2_), 7.24 (d, 4H, *J* = 8 Hz, Ar-**H**), 8.21 (d, 4H, *J* = 8 Hz, Ar-**H**), 8.43 (dd, 0.25H, *J* = 8 Hz, Ar-**H**), 8.54 (dd, 0.75H, *J* = 8 Hz, Ar-**H**), 8.58 (d, 0.25H, *J* = 4 Hz, Ar-**H**), 8.75 (d, 1.75H, *J* = 8 Hz, Ar-**H**), 8.80 (s, 0.2H, **H**-C=N), 8.87 (s, 0.8H, **H**-C=N), 9.12 (d, 1H, *J* = 4 Hz, Ar-**H**), 9.32 (d, 1H, *J* = 8 Hz, Ar-**H**), 9.41 (d, 0.25H, *J* = 4 Hz, Ar-**H**), 9.42 (d, 1.75H, *J* = 8 Hz, Ar-**H**), 9.51 (s, 0.25H, Ar-**H**), 9.57 (s, 0.75H, Ar-**H**), 13.15 (s, 0.25H, CON**H**), 13.29 (s, 0.75H, CON**H**). ^13^C NMR (100 MHz, DMSO-*d*_6_): δ_C_ = 66.67; 66.73 (2×N**C**H_2_), 113.89; 125.76; 126.44; 126.34; 126.87; 129.34; 131.55; 134.79; 144.23; 144.54; 145.23; 146.54; 146.23; 146.87 (Ar-**C**), 160.04, 164.78 (**C**=N, **C**=O), 189.10, 189.33 (2×CH_2_**C**=O).

- ***1-(2-(4-trifloromethylphenyl)-2-oxoethyl)-3-((2-(1-(2-(4-trifloromethylphenyl)-2-oxoethyl)pyridin-1-ium-4-carbonyl)hydrazono)methyl)pyridin-1-iumbromide*, 3(b).**

Yield: 91 %. ^1^H NMR (400 MHz, DMSO-*d*_6_): δ_H_ = 6.78 (d, 4H, *J* = 8 Hz, 2×NC**H**_2_), 7.24 (d, 4H, *J* = 8 Hz, Ar-**H**), 8.21 (d, 4H, *J* = 8 Hz, Ar-**H**), 8.43 (dd, 0.25H, *J* = 8 Hz, Ar-**H**), 8.54 (dd, 0.75H, *J* = 8 Hz, Ar-**H**), 8.58 (d, 0.25H, *J* = 4 Hz, Ar-**H**), 8.75 (d, 1.75H, *J* = 8 Hz, Ar-**H**), 8.80 (s, 0.2H, **H**-C=N), 8.87 (s, 0.8H, **H**-C=N), 9.12 (d, 1H, *J* = 4 Hz, Ar-**H**), 9.32 (d, 1H, *J* = 8 Hz, Ar-**H**), 9.41 (d, 0.25H, *J* = 4 Hz, Ar-**H**), 9.42 (d, 1.75H, *J* = 8 Hz, Ar-**H**), 9.51 (s, 0.25H, Ar-**H**), 9.57 (s, 0.75H, Ar-**H**), 13.15 (s, 0.25H, CON**H**), 13.29 (s, 0.75H, CON**H**). ^13^C NMR (100 MHz, DMSO-*d*_6_): δ_C_ = 66.67; 66.73 (2×N**C**H_2_), 113.89; 125.76; 126.44; 126.34; 126.87; 129.34; 131.55; 134.79; 144.23; 144.54; 145.23; 146.54; 146.23; 146.87 (Ar-**C**), 160.04, 164.78 (**C**=N, **C**=O), 189.10, 189.33 (2×CH_2_**C**=O).

1. **General procedure for the synthesis of P_ILs_ 4(a,b)**

Potassium tetrafluoroborate (2.5 mmol) was added to a solution of P_ILs_ **3(a,b)** (1 mmol) in acetonitrile (20 ml), and the reaction mixture was refluxed for 16 hours. After cooling, the product was filtered for the desired dicationic liquids **4 (a,b).**

- ***1-(2-(4-bromophenyl)-2-oxoethyl)-3-((2-(1-(2-(4-bromophenyl)-2-oxoethyl)pyridin-1-ium-4-carbonyl)hydrazono)methyl)pyridin-1-ium tetrafluoroborate*  (4a) (BPHP)**.

Physical state: solid (powder); color: pale yellow; yield: 96 %; melting point: 86-88 °C. ^1^H NMR (400 MHz, DMSO-*d*_6_): δ_H_ = 6.51 (s, 2H, NC**H**_2_), 6.61 (s, 2H, *J* = 8.0 Hz, 2×NC**H**_2_), 7.71-7.75 (m, 4H, Ar-**H**), 7.96-8.16(m, 5H, Ar-**H**), 8.25 (d, 0.75H, *J* = 8 Hz, Ar-**H**), 8.27 (d, 0.25H, *J* = 8 Hz, Ar-**H**), 8.37(s, 0.25H, **H**-C=N), 8.55(dd, 2H, *J*_1_= 8.0 Hz, *J_2_* = 4.0 Hz Ar-**H**), 8.69 (d, 1.5 H, *J* = 8 Hz, Ar-**H**), 8.78(s, 0.75H, **H**-C=N), 8.95 (d, 0.5 H, *J* = 8.0 Hz, Ar-**H**), 9.03 (d, 1.5 H, *J* = 8 Hz, Ar-**H**), 9.17 (d, 0.5 H, *J* = 8 Hz, Ar-**H**), 9.25 (d, 1.5 H, *J* = 8 Hz, Ar-**H**), 13.34 (s, 0.250H, CON**H**), 13.42 (s, 0.75 H, CON**H**). ^13^C NMR (100 MHz, DMSO-*d*_6_): δ_C_ =66.17; 66.81 (2×N**C**H_2_), 114.97; 115.01; 125.18; 126.81; 126.84; 131.30; 131.36; 145.09; 147.29; 147.94; 149.89; 160.59; 164.84; 164.90; (**C**=N, **C**=O), 189.14, 189.51 (2×CH_2_**C**=O).

- ***1-(2-(4-trifloromethylphenyl)-2-oxoethyl)-3-((2-(1-(2-(4-trifloromethylphenyl)-2 oxoethyl)pyridin-1-ium-4-carbonyl)hydrazono)methyl)pyridin-1-ium tetrafluoroborate* (4b) (TFPHP)**.

Physical state: solid (powder); color: reddish brown; yield: 97 %; melting point: 92-95 °C. ^1^H NMR (400 MHz, DMSO-*d*_6_): δ_H_ = 6.44 (d, 4H, *J* = 8.0 Hz, NC**H**_2_), 6.54 (d, 2H, *J* = 8.0 Hz, 2×NC**H**_2_), 7.17 (dd, 4H, *J*_1_= 8.1 Hz, *J_2_* = 4.0 Hz, Ar-**H**), 7.99-8.04(m, 5H, Ar-**H**), 8.25 (d, 0.6H, *J* = 8 Hz, Ar-**H**), 8.25 (d, 0.6H, *J* = 8 Hz, Ar-**H**), 8.36(s, 0.3H, **H**-C=N), 8.53(dd, 2H, *J*_1_= 8.0 Hz, *J_2_* = 4.0 Hz Ar-**H**), 8.67 (d, 1.75H, *J* = 8 Hz, Ar-**H**), 8.75(s, 0.7H, **H**-C=N), 8.94 (d, 0.6H, *J* = 8.0 Hz, Ar-**H**), 9.03 (d, 1.4 H, *J* = 8 Hz, Ar-**H**), 9.16 (d, 0.6 H, *J* = 8 Hz, Ar-**H**), 9.24 (d, 1.4 H, *J* = 8 Hz, Ar-**H**), 13.33 (s, 0.30H, CON**H**), 13.39 (s, 0.6 H, CON**H**).^13^C NMR (100 MHz, DMSO-*d*_6_): δ_C_ = 66.46; 66.08 (2×N**C**H_2_), 124.95; 125.26; 126.90; 127.67; 129.88; 129.90; 130.67; 130.78; 132.82; 132.86; 140.08; 140.18; 145.08; 147.28; 147.14; 147.49; 150.03; 160.57; (**C**=N, **C**=O), 190.14, 190.50 (2×CH_2_**C**=O).


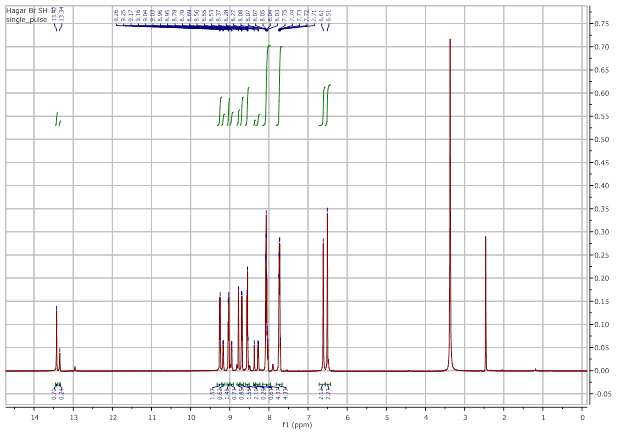


**Sup.Fig 1**: ^1^ H NMR of compound **BPHP**


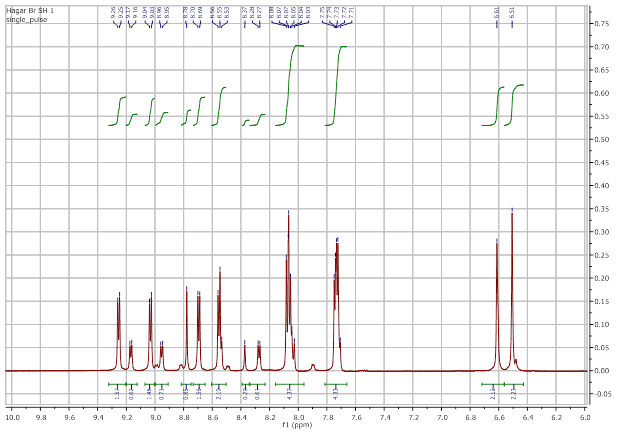


**Sup.Fig 2**: ^1^ H NMR of compound **BPHP**


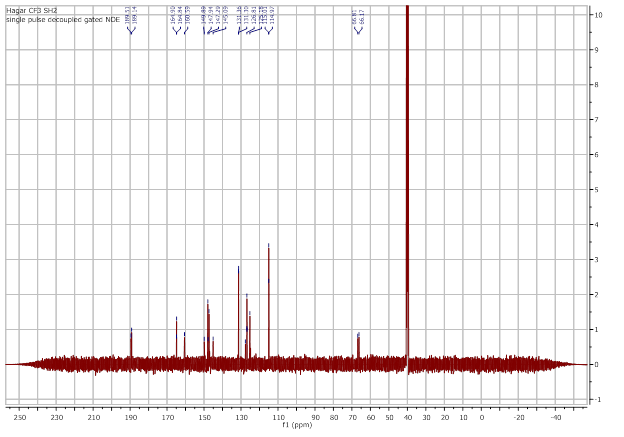


**Sup.Fig 3**: C^13^ NMR of compound **BPHP**


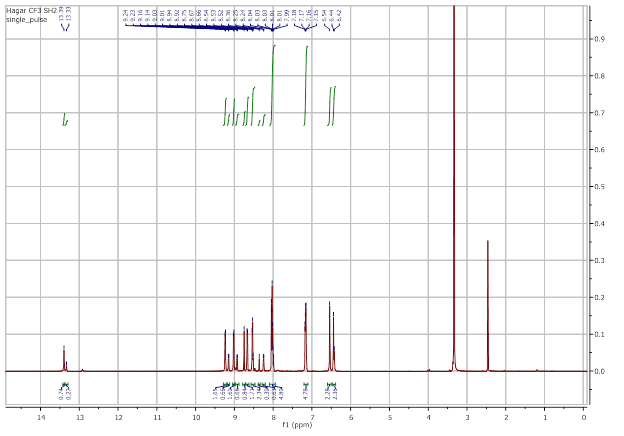


**Sup.Fig 4**: ^1^ H NMR of compound **TFPHP**


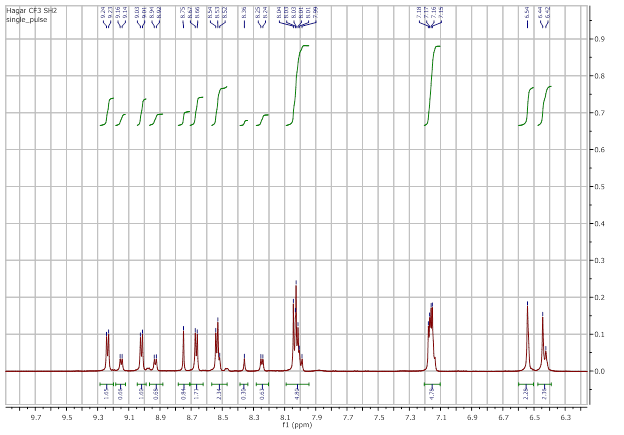


**Sup.Fig 5**: ^1^ H NMR of compound **TFPHP**


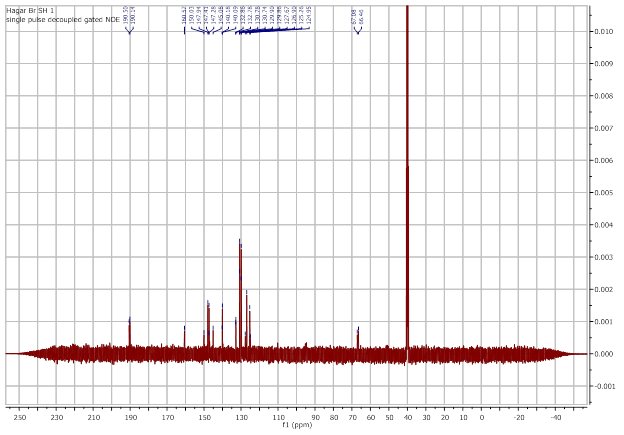


**Sup.Fig 6**: C^13^ NMR of compound **TFPHP**

Sup.Table 1: Fukui indices, local dual descriptors and Mulliken charges of different atoms of BPHP in the gas phase.

Sup.Table 2: Fukui indices, local dual descriptors and Mulliken charges of different atoms of TFPHP in the gas phase.

Sup.Table 3: Fukui indices, local dual descriptors and Mulliken charges of different atoms of BPHP in aqueous phase.

Sup.Table 4: Fukui indices, local dual descriptors and Mulliken charges of different atoms of TFPHP in aqueous phase.
